# Host Bias in Diet-Source Microbiome Transmission in Wild Cohabitating Herbivores: New Knowledge for the Evolution of Herbivory and Plant Defense

**DOI:** 10.1128/spectrum.00756-21

**Published:** 2021-08-18

**Authors:** Lifeng Zhu, Yongyong Zhang, Xinyuan Cui, Yudong Zhu, Qinlong Dai, Hua Chen, Guoqi Liu, Ran Yao, Zhisong Yang

**Affiliations:** a College of Life Sciences, Nanjing Norma University, Nanjing, China; b Sichuan Liziping National Nature Reserve, Shimian, China; c Mingke Biotechnology Co., Ltd., Hangzhou, China; d Sichuan Academy of Giant Panda, Chengdu, China; e Shimian Research Center of Giant Panda Small Population Conservation and Rejuvenation, Shimian, China; University of Minnesota

**Keywords:** diet-source microbiome transmission, gut microbiomes, wild herbivores, host-plant interaction

## Abstract

It is commonly understood that dietary nutrition will influence the composition and function of the animal gut microbiome. However, the transmission of organisms from the diet-source microbiome to the animal gut microbiome in the natural environment remains poorly understood, and elucidating this process may help in understanding the evolution of herbivores and plant defenses. Here, we investigated diet-source microbiome transmission across a range of herbivores (insects and mammals) living in both captive and wild environments. We discovered a host bias among cohabitating herbivores (leaf-eating insects and deer), where a significant portion of the herbivorous insect gut microbiome may originate from the diet, while in deer, only a tiny fraction of the gut microbiome is of dietary origin. We speculated that the putative difference in the oxygenation level in the host digestion systems would lead to these host biases in plant-source (diet) microbiome transmission due to the oxygenation living condition of the dietary plant’s symbiotic microbiome.

**IMPORTANCE** We discovered a host bias among cohabitating herbivores (leaf-eating insects and deer), where a significant portion of the herbivorous insect gut microbiome may originate from the diet, while in deer, only a tiny fraction of the gut microbiome is of dietary origin. We speculated that the putative difference in the oxygenation level in the host digestion systems would lead to these host biases in plant-source (diet) microbiome transmission due to the oxygenation living condition of the dietary plant's symbiotic microbiome. This study shed new light on the coevolution of herbivory and plant defense.

## INTRODUCTION

Host phylogeny and diet are two major factors shaping the animal gut microbiome (1–4). It is also commonly understood that dietary nutrition influences the composition and function of the animal gut microbiome (5, 6). In nature, herbivorous animal food sources (e.g., plants) harbor a number of the symbiotic microbiome (7, 8). This leads to the question of whether these diet-source microbiomes would survive in the animal intestinal tract and become an integrated part of the animal gut microbiome. The development of the animal gut microbiome includes vertical (e.g., from mothers to offspring) and horizontal (e.g., from the living environment to individuals) transmission (9–14). However, how diet-source microbiome transmission occurs in nature is still poorly understood.

Our previous study on the Père David’s deer (Elaphurus davidianus; Chinese milu) gut microbiome found that the enzymes *natA* and *natB*, involved in the sodium transport system, were enriched in the gut microbiome in a translocated population (15). This was possibly due to their high salt diet, which is primarily composed of Spartina alterniflora and Phragmites australis (15), the most abundant plants in the wild translocation site (coastal area) (see Fig. S1 in the supplemental material) (16–18). We also observed abundant insect life (e.g., grasshoppers and locusts) in the S. alterniflora and P. australis habitat of this area, of which the main dietary sources were also these two highly abundant plants. Thus, this area was a suitable field site to test diet-source microbiome transmission in nature for the following reasons: (i) the plant community in the area is straightforward, (ii) the major dietary plants are the same for both insects (e.g., grasshoppers and locusts) and mammals, and (iii) the sympatric distribution of the insects (e.g., grasshoppers and locusts) and milu.

Insects (e.g., grasshoppers and locusts) belong to invertebrates, and the milu are vertebrates. The oxygenation of insect guts varies from fully aerobic to anaerobic (19), while the milu, a type of ruminant, harbors an anaerobic gut (20). Plants harbor a number of symbiotic microbiomes, the majority of which exist in an aerobic environment (7, 8). Therefore, we hypothesized in this study that there would be a bias in the transmission of diet-source microbiome between the sympatric herbivorous insects (e.g., grasshoppers and locusts) and mammal (e.g., milu) in the wild habitat and that the gut microbiome of grasshoppers and locusts would harbor a higher proportion of diet-source microorganisms, while the milu would harbor a much lower proportion. Moreover, we also investigated diet-source microbiome transmission among other herbivores (insects and mammals) living in both captive conditions and in the natural environment.

## RESULTS AND DISCUSSION

**FIG 1:**
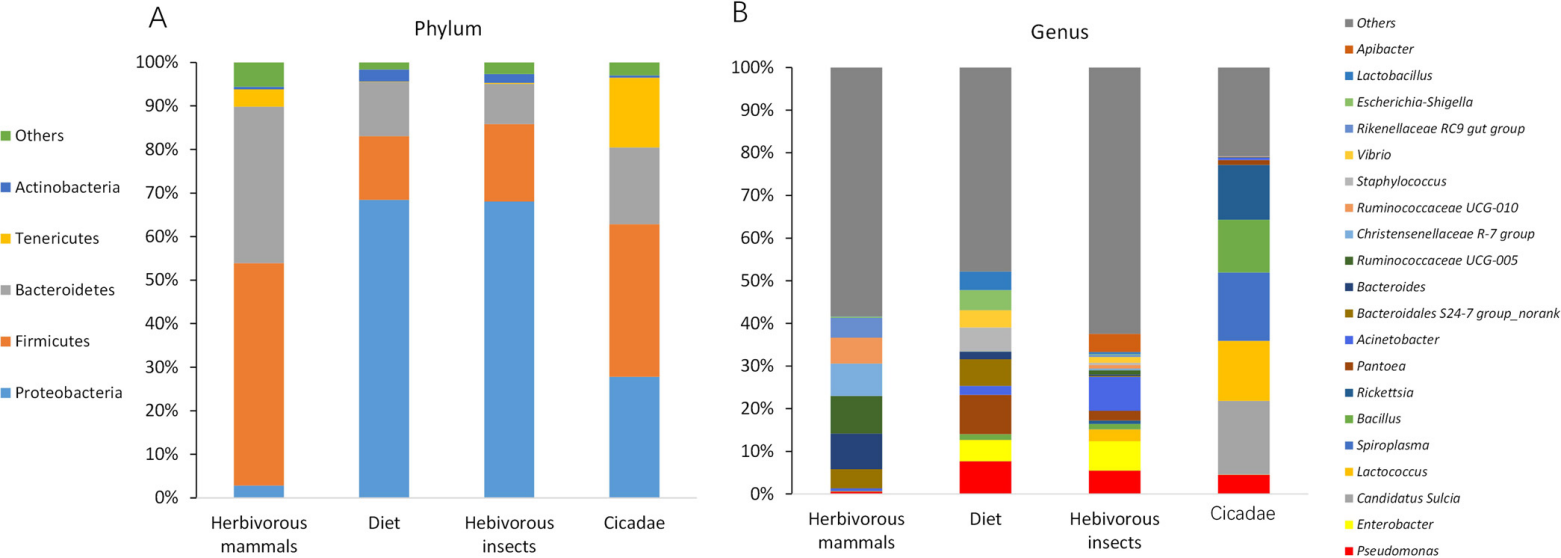
The dominant microbial groups in diet and herbivores of this study. (A) Phylum level. (B) Genus level. Herbivorous insects included 43 pooled samples from the following: leaf-eating grasshoppers, locusts, and Pyralidae. Herbivorous mammals included 89 samples from the following: Père David’s Deer (milu), musk deer, and rabbits. Diet included 81 dietary plant samples and five forage samples. Cicadas were of the sap-sucking type.

We obtained 16S rRNA gene data from 285 samples (Table 1). The main phyla in the diet (81 dietary plant samples and five forage samples) included *Proteobacteria*, *Firmicutes*, and *Bacteroidetes*. The dominant phyla in the gut microbiomes of herbivorous insects (43 pooled samples from leaf-eating grasshoppers, locusts, and Pyralidae) included *Proteobacteria*, *Firmicutes*, and *Bacteroidetes*, while in the sap-sucking cicadas, the main phyla included *Proteobacteria*, *Firmicutes*, *Bacteroidetes*, and *Tenericutes*. The dominant phyla in herbivorous mammals (89 samples including Père David's deer [milu], musk deer, and rabbits) included *Firmicutes* and *Bacteroidetes* (Fig. 1A).

**TABLE 1:** Sample information in this study

Sample name	Sample size	Sample type	Location	Living style
Red rabbit	29	Feces	Dayi	Captive
Dietary food-red rabbit	2	Forage	Dayi	
Musk deer	44	Feces	Dujiangyan	Captive
Dietary food-musk deer	28	Plant and forage	Dujiangyan	
Grasshoppers and Locusts	27^*a*^	Gut contents	Dafeng	Wild
Père David's Deer	45	Feces	Dafeng	Wild
Dietary food in Dafeng	34	Plant	Dafeng	
Cicadas	16^*a*^	Gut contents	Liyang	Wild
Pyralidae	6^*a*^	Gut contents	Liyang	Wild
Dietary food in Liyang	5	Plant	Liyang	
Cicadas	22^*a*^	Gut contents	Xuzhou	Wild
Dietary food-Cicadas	6	Plant	Xuzhou	
Pyralidae	10^*a*^	Gut contents	Anji	Wild
Dietary food-Pyralidae	11	Plant	Anji	

a Due to the small amount of gastrointestinal content available in a single insect, the gut contents from five individuals were pooled as one insect sample for DNA extraction, and the majority of the 81 insect samples were pooled samples.

**FIG 2:**
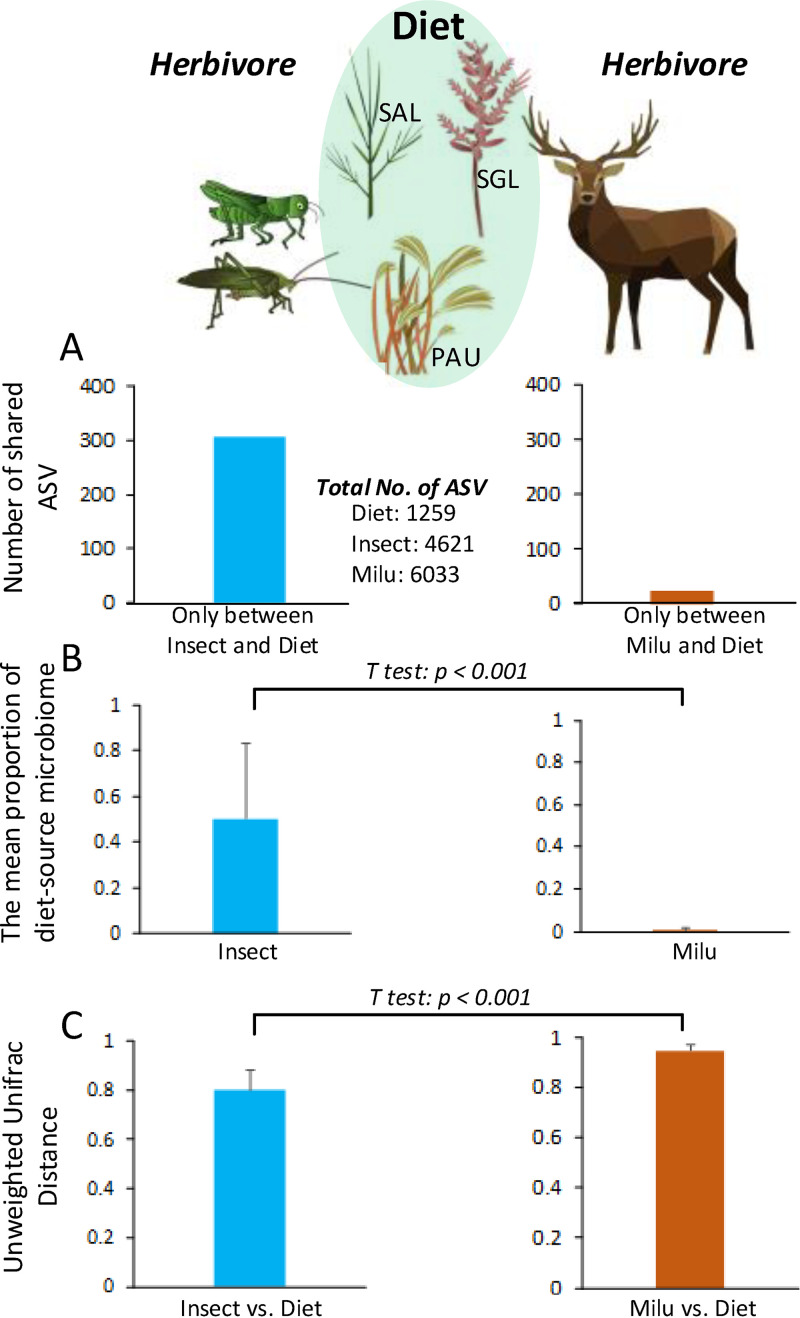
The diet-source microbiome transmission in wild cohabitating herbivores. (A) ASVs (amplicon sequence variants) shared only between the gut microbiome herbivorous insects (leaf-eating grasshoppers and locusts) and dietary plants (left) and only between the gut microbiome of milu and dietary plants (right). (B) Mean proportion of diet-source microbiome in herbivorous insect gut microbiome (left) and milu gut microbiome (right). (C) Mean pairwise unweighted UniFrac distance between insect and diet samples (left) or between milu and diet samples (right). Nonparametric *t* test was used to test the significance of the difference. SAL, Spartina alterniflora; PAU, Phragmites australis; SGL, Suaeda glauca.

Moreover, the dominant genera in the dietary samples included *Pantoea*, *Pseudomonas*, *Enterobacter*, and *Acinetobacter*. The dominant genera in herbivorous insects included *Pseudomonas*, *Enterobacter*, *Acinetobacter*, *Pantoea*, and *Lactococcus*. In contrast, the dominant genera in herbivorous mammals included *Ruminococcaceae* UCG 005, *Ruminococcaceae* UCG 010, *Christensenellaceae* R7 group, and *Bacteroides*, and in sap-sucking cicadas, they included “*Candidatus* Sulcia,” *Spiroplasma*, *Lactococcus*, *Rickettsia*, *Bacillus*, and *Pseudomonas* (Fig. 2B). Thus, based on the microbiome composition, we found some common features (relatively high proportion of *Proteobacteria*, *Pseudomonas*, *Enterobacter*, and *Pantoea*) between the herbivorous insect gut microbiome and diet-source microbiome. Moreover, permutational multivariate analysis of variance (PERMANOVA) using unweighted UniFrac distance showed a significant dissimilarity (*P* < 0.01) in the gut microbiome community among these host groups.

### Herbivorous insects harbored a significantly higher proportion of diet-source microbiome in their gut microbiome than sympatric deer.

Although the total number of amplicon sequence variants (ASVs) in the milu gut microbiome was largest (6,033 ASVs in milu, 4,621 in insects, and 1,259 in dietary plants), we found that the gut microbiome of herbivorous insects harbored a greater number of organisms originating from their diet than that harbored by herbivorous milu (Fig. 2). For example, the shared ASVs in the gut microbiome between herbivorous insects and dietary plants was 305 (see Table S2 in the supplemental material) compared with 23 shared (see Table S3 in the supplemental material) between milu and dietary plants (Fig. 2A; see also Fig. S2 in the supplemental material). The ASVs shared between herbivorous insects and dietary plants primarily belonged to the *Proteobacteria* (*Gammaproteobacteria* including *Pantoea*, *Kluyvera*, *Escherichia*, and *Shigella* from the *Enterobacteriales*; *Acinetobacter* and *Pseudomonas* from the *Pseudomonadales*; and *Pseudoalteromonas* from the *Alteromonadales*) and *Firmicutes* (*Bacillus* and *Exiguobacterium*) (see Fig. S3 in the supplemental material). The proportion of the putative diet-source microbiome was significantly higher in the insect gut microbiome (0.50 ± 0.328) than in the milu gut microbiome (0.00 ± 0.011) (nonparametric *t* test, *P* < 0.001) (Fig. 2B). This finding indicated that a high proportion of the gut microbiome of herbivorous insects might originate from the symbiotic microbiome of their dietary plants. Finally, the pairwise unweighted UniFrac distance was significantly lower between the insect gut microbiomes and diet-source microbiome than that between the milu gut microbiome and diet-source microbiome (nonparametric *t* test, *P* < 0.001) (Fig. 2C). This finding indicated a high similarity between the insect gut microbiome communities and the dietary plant symbiotic microbiome.

**FIG 3:**
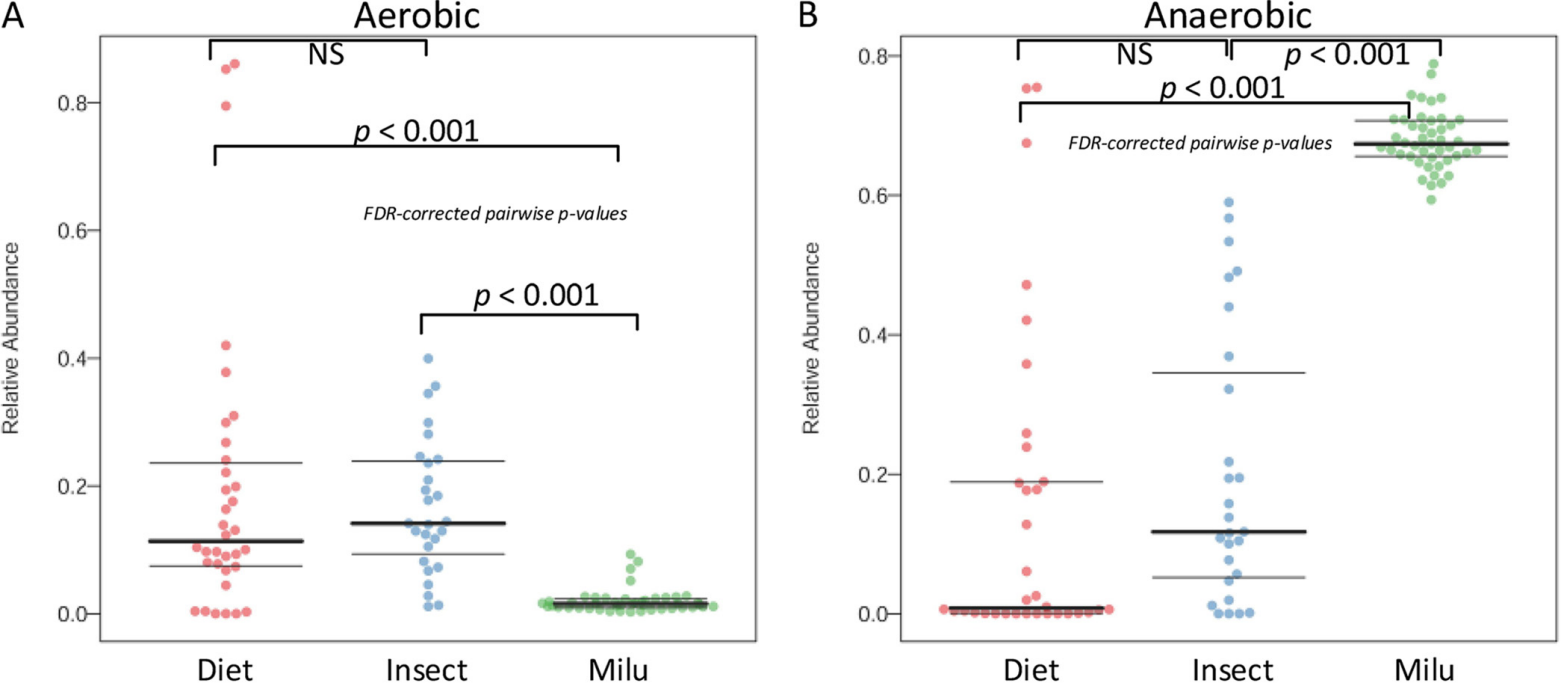
The putative phenotype in the microbiome in this study. (A) Proportion of putative aerobic. (B) Proportion of putative anaerobic. The Mann-Whitney-Wilcoxon test was used to assess the hypothesis of whether the proportion of aerobic microbiome in either insect gut microbiome or diet-source microbiome was significantly higher than that in the milu gut microbiome. Herbivorous insects included leaf-eating grasshoppers and locusts in the Dafeng region. Diet included dietary plants for the insects and milu in the Dafeng region.

Interestingly, the proportion of the aerobic microbiome in the insect gut microbiome and diet-source microbiome was significantly higher than that in the milu gut microbiome (Fig. 3A), while the opposite trend was observed for the respective anaerobic microbiomes (Fig. 3B). We speculated that this could be due to the level of oxygenation in these animal guts (7, 8, 19, 20), and this would explain the survival of the dietary source microbiome in herbivorous insect guts. Therefore, based on this evidence, we conclude that there is evidence for host bias in diet-source microbiome transmission among the gut microbiomes of wild cohabitating herbivores. However, there remained the question of whether there was a common pattern among other herbivorous animals.

### Host bias in diet-source microbiome transmission was confirmed across other herbivores (insects and mammals) living in captive and wild environments.

**FIG 4:**
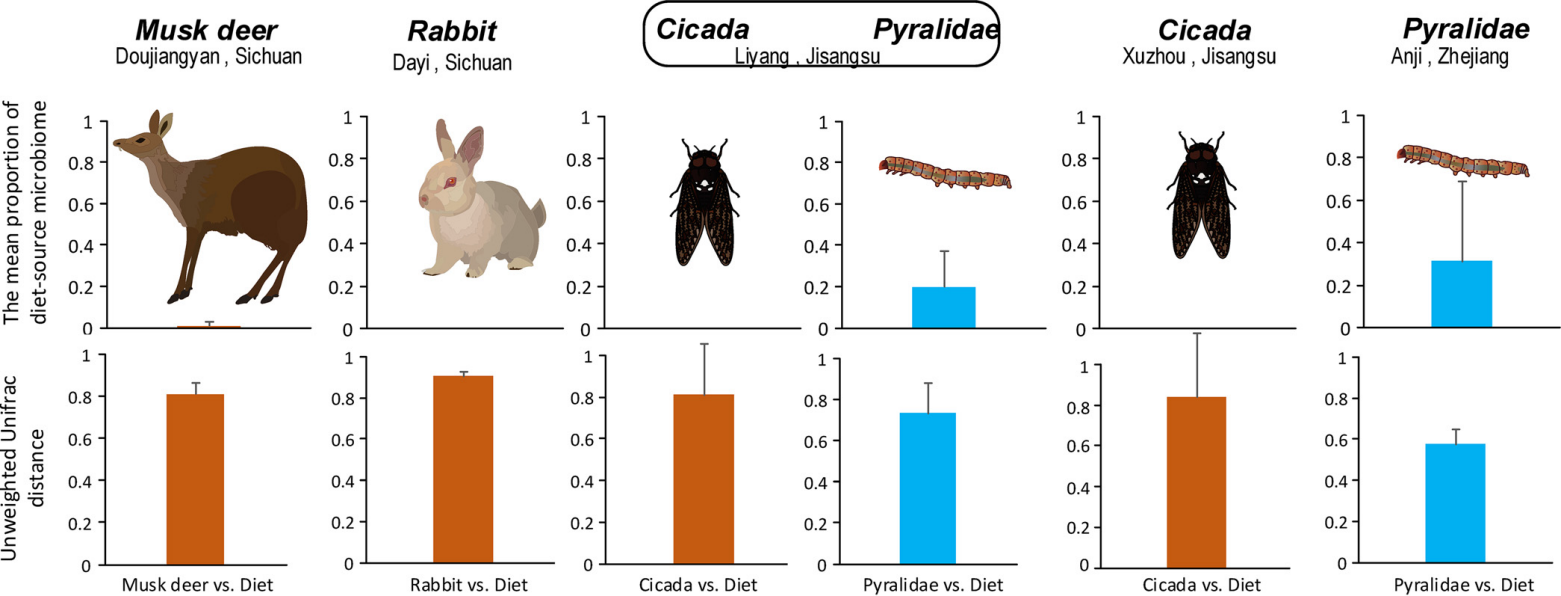
The diet-source microbiome transmission across different herbivorous animals from invertebrates (wild Pyralidae insects and cicadas) to vertebrates (captive musk deer and rabbits). The first row presents the mean proportion of diet-source microbiome in herbivorous insect and mammal gut microbiome. The second row presents the mean pairwise unweighted UniFrac distance between herbivore and diet samples. Pyralidae collected in the Liyang region were adults. Pyralidae collected in the Anji region were adults and larva.

Next, we investigated the potential for host bias across different herbivorous animals (including invertebrates [wild Pyralidae insects and cicadas] and vertebrates [captive musk deer and rabbits]) (Fig. 4). We confirmed host bias in diet-source microbiome transmission among gut microbiomes. For instance, the proportion of diet-source microbiome in herbivorous musk deer and rabbits was close to zero. In contrast, the mean proportion in the Pyralidae insects (living in two separate wild regions) was about 25% (Fig. 4). However, the proportion in cicadas, which suck juice and saps from perennial plants, from two wild regions was close to zero. The discrepancy between the Pyralidae insects and cicadas might be partially due to the different feeding habits (leaf eating versus sap sucking). The pairwise unweighted UniFrac distance between the host microbiome and dietary plant symbiotic microbiome was lower in Pyralidae insects than in other animals (including milu, rabbits, and cicadas) (Fig. 4) and further supported our findings regarding host bias in the transmission of diet-source microbiomes.

The development of the animal gut microbiome includes vertical transmission (e.g., from mothers to offspring) and horizontal transmission (9–14). Previous studies have revealed a putative connection between the host gut microbiome and diet-source microbiome (13, 21, 22). However, in nature, we showed a host bias in the diet-source microbiome. The proportion of diet-source microbiome was higher in some herbivorous insects due to the potential oxygen level in their gut. Thus, we speculated that these diet-source microbiomes would survive in the animal intestinal tract. This study uncovered new evidence that a portion of the animal gut microbiome might be derived from their diet. The amount would be host-specific, depending on the conditions present in their inner intestines.

### The implications for evolution in herbivores and plant defense.

Plant defense and optimal foraging by herbivores are typical interactions in nature (23, 24). Plant secondary metabolites (PSMs) are considered a defense against pathogens or herbivores (both invertebrate and vertebrate) (24, 25). Herbivores utilize both behavioral methods and physiological strategies to detoxify and limit the harmful effects of PSMs (25–28). For example, in addition to the digestion of the plant primary compounds (e.g., carbohydrates, proteins, and lipids), herbivores have developed numerous enzymes (e.g., P450s, glutathione *S*-transferases [GSTs], choline/carboxylesterases [CCEs], and glucuronosyltransferases) to detoxify PSMs (25, 29–31). Some studies have found that the animal gut microbiome might be involved in the detoxification of PSMs (32, 33). Our previous studies found that *Enterobacteriales* and *Pseudomonadales* (belonging to *Proteobacteria*) in the gut microbiomes of bamboo-eating panda were associated with the detoxification of bamboo PSM (e.g., cyanide compounds) (34, 35). Spartina alterniflora, one of the major dietary plants in this study, also contains cyanide compounds (concentration, about 0.1 μg/g) (34). Many strains from the *Enterobacteriales* and *Pseudomonadales* play essential roles in biodegradation and detoxification of PSMs (36–39). For example, many *Pantoea* strains are plant pathogens (40, 41), some of which can degrade specific PSMs (tannic acid) and environmental polyphenols (42–45).

Interestingly, the main organisms transferred from the dietary plant symbiotic microbiome into herbivorous insects included *Enterobacteriales* and *Pseudomonadales* (Table 2). Here, we didn't make the functional analysis for these microbiomes. The functional evaluation in the closed phylogenetic microbiome of *Enterobacteriales* and *Pseudomonadales* was presented in previously published studies (36–39). Thus, we could only speculate that the adaptation to host plants by herbivorous grasshoppers and locusts in this study might be associated with their gut microbiome acquiring organisms from the dietary plant symbiotic microbiome. Moreover, typical herbivorous mammals (e.g., ruminant deer and rabbits) harbored a low (close to zero) proportion of diet-source microbiome. While, as previously stated, one of the main functions of their gut microbiome (high abundance of *Ruminococcaceae*) is to digest primary plant compounds (e.g., celluloses, hemicellulose, and proteins) (46, 47), they also play an important role in the detoxification of PSMs (32, 33). Compared to the herbivorous insects in this study, we speculated that those typical herbivorous mammals (e.g., milu, musk deer, and rabbits) might have evolved a different mechanism to detoxify PSMs.

**TABLE 2:** The main predicted contribution of the diet-source microbiome to gut microbiome of herbivorous insects in this study

Insect/location and DSMT^*a*^	Proportion	Taxon
Grasshopper and locust in Dafeng, Jiangsu		
DSMT1	0.0408	p__*Proteobacteria*; c__*Gammaproteobacteria*; o__*Enterobacteriales*; f__*Enterobacteriaceae*; g__*Kluyvera*; s__*Kluyvera ascorbata*
DSMT2	0.0294	p__*Proteobacteria*; c__*Gammaproteobacteria*; o__*Pseudomonadales*; f__*Moraxellaceae*; g__*Acinetobacter*; s__uncultured bacterium
DSMT3	0.0247	p__*Proteobacteria*; c__*Gammaproteobacteria*; o__*Pseudomonadales*; f__*Moraxellaceae*; g__*Acinetobacter*
DSMT4	0.0206	p__*Firmicutes*; c__*Bacilli*; o__*Bacillales*; f__family XII; g__*Exiguobacterium*
DSMT5	0.0159	p__*Proteobacteria*; c__*Gammaproteobacteria*; o__*Vibrionales*; f__*Vibrionaceae*; g__*Vibrio*; s__*Vibrio fluvialis*
DSMT6	0.0157	p__*Proteobacteria*; c__*Gammaproteobacteria*; o__*Enterobacteriales*; f__*Enterobacteriaceae*; g__*Enterobacter*
DSMT7	0.0154	p__*Proteobacteria*; c__*Gammaproteobacteria*; o__*Enterobacteriales*; f__*Enterobacteriaceae*; g__*Pantoea*
DSMT8	0.0153	p__*Proteobacteria*; c__*Gammaproteobacteria*; o__*Pseudomonadales*; f__*Moraxellaceae*; g__*Acinetobacter*
DSMT9	0.0108	p__*Proteobacteria*; c__*Gammaproteobacteria*; o__*Xanthomonadales*; f__*Xanthomonadaceae*; g__*Pseudoxanthomonas*; s__uncultured bacterium
DSMT10	0.0087	p__*Firmicutes*; c__*Bacilli*; o__*Bacillales*; f__Family XII; g__*Exiguobacterium*; s__*Exiguobacterium indicum*
Pyralidae insects in Liyang, Jiangsu		
DSMT6	0.1516	p__*Proteobacteria*; c__*Gammaproteobacteria*; o__*Enterobacteriales*; f__*Enterobacteriaceae*; g__*Enterobacter*
DSMT11	0.0236	p__*Proteobacteria*; c__*Gammaproteobacteria*; o__*Enterobacteriales*; f__*Enterobacteriaceae*; g__*Pantoea*
DSMT12	0.0154	p__*Proteobacteria*; c__*Gammaproteobacteria*; o__*Enterobacteriales*; f__*Enterobacteriaceae*; g__*Serratia*
DSMT13	0.0025	p__*Proteobacteria*; c__*Gammaproteobacteria*; o__*Enterobacteriales*; f__*Enterobacteriaceae*; g__*Klebsiella*; s__uncultured bacterium
DSMT14	0.0023	p__*Proteobacteria*; c__*Gammaproteobacteria*; o__*Xanthomonadales*; f__*Xanthomonadaceae*; g__ *Stenotrophomonas*; s__uncultured bacterium
DSMT15	0.0015	p__*Proteobacteria*; c__*Gammaproteobacteria*; o__*Pseudomonadales*; f__*Pseudomonadaceae*; g__*Pseudomonas*
DSMT16	0.0011	p__*Proteobacteria*; c__*Gammaproteobacteria*; o__*Xanthomonadales*; f__*Xanthomonadaceae*; g__*Stenotrophomonas*; s__uncultured bacterium
DSMT17	0.0006	p__*Proteobacteria*; c__*Gammaproteobacteria*; o__*Enterobacteriales*; f__*Enterobacteriaceae*; g__*Enterobacter*
DSMT18	0.0005	p__*Actinobacteria*; c__*Actinobacteria*; o__*Micrococcales*; f__*Microbacteriaceae*; g__*Microbacterium*
DSMT19	0.0001	p__*Firmicutes*; c__*Bacilli*; o__*Lactobacillales*; f__*Lactobacillaceae*; g__*Lactobacillus*
Pyralidae insects in Anji, Zhejiang		
DSMT17	0.1050	p__*Proteobacteria*; c__*Gammaproteobacteria*; o__*Enterobacteriales*; f__*Enterobacteriaceae*; g__*Enterobacter*
DSMT15	0.0896	p__*Proteobacteria*; c__*Gammaproteobacteria*; o__*Pseudomonadales*; f__*Pseudomonadaceae*; g__*Pseudomonas*
DSMT11	0.0234	p__*Proteobacteria*; c__*Gammaproteobacteria*; o__*Enterobacteriales*; f__*Enterobacteriaceae*; g__*Pantoea*
DSMT16	0.0180	p__*Proteobacteria*; c__*Gammaproteobacteria*; o__*Xanthomonadales*; f__*Xanthomonadaceae*; g__*Stenotrophomonas*; s__uncultured bacterium
DSMT18	0.0157	p__*Proteobacteria*; c__*Gammaproteobacteria*; o__*Pseudomonadales*; f__*Moraxellaceae*; g__*Acinetobacter*; s__uncultured bacterium
DSMT6	0.0096	p__*Proteobacteria*; c__*Gammaproteobacteria*; o__*Enterobacteriales*; f__*Enterobacteriaceae*; g__*Enterobacter*
DSMT19	0.0081	p__*Proteobacteria*; c__*Gammaproteobacteria*; o__*Pseudomonadales*; f__*Pseudomonadaceae*; g__*Pseudomonas*
DSMT20	0.0061	p__*Proteobacteria*; c__*Alphaproteobacteria*; o__*Rhizobiales*; f__*Brucellaceae*; g__*Ochrobactrum*
DSMT21	0.0043	p__*Proteobacteria*; c__*Betaproteobacteria*; o__*Burkholderiales*; f__*Comamonadaceae*; g__*Delftia*
DSMT12	0.0034	p__*Proteobacteria*; c__*Gammaproteobacteria*; o__*Enterobacteriales*; f__*Enterobacteriaceae*; g__*Serratia*

a DSMT, diet-source microbiome transmission.

Therefore, we speculated that the simplest and superior way for the adaptation to host-plant interactions might be to harbor select organisms from the dietary plant’s symbiotic microbiome beyond host generated enzymes. In addition, here, we didn't sample all of the animals in the environment or test the hypothesis in all of the animals. Therefore, the current findings are limited to the animals that we investigated (e.g., grasshopper, locust, cicada, Pyralidae, milu, and musk deer).

### Conclusion.

Here, we uncovered host bias in the diet-source microbiome of cohabitating herbivores and show that a variable portion of the normal animal gut microbiome might be derived from dietary sources. We speculated that the putative difference in the oxygenation level in the host digestion systems would lead to these host-biases in plant-source (diet) microbiome transmission due to the oxygenation living condition of the dietary plant’s symbiotic microbiome. Furthermore, we suggest that future work focuses on functional evaluation of the colonization of the dietary plant symbiotic microbiome in herbivorous insects and tests whether they play an important role in detoxifying and decreasing the negative effects of PSMs.

## MATERIALS AND METHODS

### Sample collection.

We collected the sympatric insects (grasshoppers and locusts) and milu fecal samples in the wetlands of Dafeng (Jiangsu Province) (Table 1; see also Table S1 in the supplemental material). The leaves and stems of the major dietary plants were collected at the same time. We also collected samples from captive and wild herbivores (insects and mammals) (Table 1; Table S1). For example, in the bamboo forest in Liyang city, we collected cicadas, Pyralidae, and dietary bamboo samples. In the bamboo forest in Anji city, we collected Pyralidae and dietary bamboo samples. In the forest in Xuzhou city, we collected cicadas and dietary plant samples. In the rabbit breeding center in Dayi city, we collected fresh feces for each rabbit individual and dietary forage samples. During sampling, each cage only has one rabbit; thus, we could collect fresh feces. In the musk deer breeding center in Dujiangyan city, we collected fresh feces for each musk deer individual and dietary plant samples. Each cage only has one musk deer individual, so we could collect fresh feces for each individual. The fresh feces were placed into 15-ml sterile tubes. Because the deer live alone, the feces could be marked to each individual. The forage and plant samples were reserved in aseptic plastic bags.

All instruments and materials were sterilized prior to sampling. The insects were frozen after sampling and then shipped to the lab on dry ice. Each insect was successfully dissected. We cut the whole gut (including the contents) using the dissecting microscope and then transferred the whole gut into 2-ml aseptic centrifuge tubes. Due to the small amount of gastrointestinal content available in a single insect, the gut contents from five individuals were pooled as one insect sample for DNA extraction, and the majority of the 81 insect samples were pooled samples. In this study, in total, we collected 86 samples from the diet (including 81 dietary plant samples and five forage samples) (Table S1). Fresh feces were collected from the herbivorous mammals. All plant samples and fresh feces were frozen (−20°C) upon collection and then shipped on dry ice to the laboratory for analysis.

### DNA extraction and metagenomic sequencing.

The FastDNA spin kit for feces (MP, OH, USA) was used to extract microbial DNA from the gut contents and fecal samples once they were thawed at room temperature. The FastDNA spin kit for soil (MP, OH, USA) was used to extract microbial DNA from the plant samples. We used primers 515F (5′-GTGCCAGCMGCCGCGGTAA-3′) and 806R (5′-GACTACHVGGGTWTCTAAT-3′) to amplify the V4 region of the bacterial 16S rRNA gene. The thermocycling reaction conditions were as follows: 95°C for 5 min and 35 cycles of 95°C for 30 s, 55°C for 30 s, and 72°C for 45 s, with a final extension step at 72°C for 10 min. High-throughput sequencing of amplicons was performed using the Illumina MiSeq platform. Sequencing was performed by Mingke Biotechnology Co., Ltd. (Hangzhou, China).

### 16S rRNA gene-based sequence analysis.

We performed quality control (e.g., demultiplex and denoise) and taxon classification (against the SILVA132 database) in QIIME 2 (48). ASV (amplicon sequence variant) abundance tables for downstream analysis were obtained using QIIME 2. We chose to rarefy our sampling depth at 19,902 reads per sample to equalize the sampling depth across all samples. We used four strategies to investigate diet-source microbiome transmission in the herbivore gut microbiomes.

First, to assess which herbivores possessed a gut microbiome that was more similar to the diet source, we estimated the number of ASVs shared between them. Second, SourceTracker (49) was used to assess the contribution (microbiome transmission) of the diet-source microbiomes to the herbivore samples. For example, in each sampling region, the diet was treated as the source, and the herbivorous insects (groups) or mammals were treated as sinks. For some sampling regions in this study, sap-sucking (cicadas) rather than leaf-feeding insects were sampled, and thus, we treated them as a different insect group.

The nonparametric *t* test was used to analyze the differences in the contribution of the diet-source microbiome in the gut microbiome between the herbivorous insect and mammal samples. Third, we calculated the pairwise unweighted UniFrac among these samples in QIIME 2 (48). Then, we gauged the mean distance between the herbivorous animal and diet samples. Nonparametric *t* test was used to analyze the differences in the mean pairwise distance between the herbivorous insect (versus diet) and mammal (versus diet). This could show the similarity in the microbiome community between the herbivore and diet samples. Fourth, BugBase was used to classify samples into different microbial groups. Group classifications included aerobic and anaerobic bacteria (50). We tested (Mann-Whitney-Wilcoxon tests) the hypothesis of whether the proportion of aerobic microorganisms in either insect gut microbiome or diet-source microbiome was significantly higher than that in the milu gut microbiome.

In addition, SourceTracker (49) was also used to assess the contribution (microbiome transmission) of the diet-source microbiomes to other herbivorous insects (e.g., Pyralidae and cicadas) and the herbivorous mammals (e.g., musk deer and red rabbits) (Table 1). For example, in each sampling region, the dietary food was treated as the source; the herbivorous insects (groups) or mammals were treated as sinks. For some sampling regions in this study, sap-sucking (cicadas) rather than leaf-feeding insects were sampled, and thus, we treated them as a different insect group.

### Data availability.

The 16S rRNA gene data have been submitted to figshare (10.6084/m9.figshare.15073920) and are public (https://figshare.com/articles/dataset/The_fasta_clean_data/15073920).
