# Supplementary material for: Host Bias in Diet-Source Microbiome Transmission in Wild Cohabitating Herbivores: New Knowledge for the Evolution of Herbivory and Plant Defense
Source: Microbiol Spectr. 2021 Aug 18;9(1):10.1128/spectrum.00756-21. doi: 10.1128/spectrum.00756-21 (PMC8552726; doi:10.1128/spectrum.00756-21)
Supplement: SUPPLEMENTAL FILE 1 — Supplemental material. Download SPECTRUM00756-21_Supp_1_seq6.docx, DOCX file, 0.7 MB [file spectrum00756-21_supp_1_seq6.docx]

**Supporting information**

Table S1-S3

Figure S1-S3

Table 1 The sample information in this study

| **SampleID** | **Sourcetracer** | **Sample type** | **Location** | **Collecting time** | **Living style** | **Sample name** |
| --- | --- | --- | --- | --- | --- | --- |
| 02D113 | Sink | Feces | Dayi | Oct-20 | Captive | Red rabbit |
| 05K084 | Sink | Feces | Dayi | Oct-20 | Captive | Red rabbit |
| 97D132 | Sink | Feces | Dayi | Oct-20 | Captive | Red rabbit |
| 94D428 | Sink | Feces | Dayi | Oct-20 | Captive | Red rabbit |
| 97J003 | Sink | Feces | Dayi | Oct-20 | Captive | Red rabbit |
| 83D076 | Sink | Feces | Dayi | Oct-20 | Captive | Red rabbit |
| 02D003 | Sink | Feces | Dayi | Oct-20 | Captive | Red rabbit |
| 99D038 | Sink | Feces | Dayi | Oct-20 | Captive | Red rabbit |
| 95D361 | Sink | Feces | Dayi | Oct-20 | Captive | Red rabbit |
| 9HM056 | Sink | Feces | Dayi | Oct-20 | Captive | Red rabbit |
| 1HD017 | Sink | Feces | Dayi | Oct-20 | Captive | Red rabbit |
| 99D111 | Sink | Feces | Dayi | Oct-20 | Captive | Red rabbit |
| 02D043 | Sink | Feces | Dayi | Oct-20 | Captive | Red rabbit |
| 91D051 | Sink | Feces | Dayi | Oct-20 | Captive | Red rabbit |
| 01D175 | Sink | Feces | Dayi | Oct-20 | Captive | Red rabbit |
| 94D304 | Sink | Feces | Dayi | Oct-20 | Captive | Red rabbit |
| 09501D | Sink | Feces | Dayi | Oct-20 | Captive | Red rabbit |
| 01D156 | Sink | Feces | Dayi | Oct-20 | Captive | Red rabbit |
| 01D144 | Sink | Feces | Dayi | Oct-20 | Captive | Red rabbit |
| 94D233 | Sink | Feces | Dayi | Oct-20 | Captive | Red rabbit |
| 01D216 | Sink | Feces | Dayi | Oct-20 | Captive | Red rabbit |
| 91D176 | Sink | Feces | Dayi | Oct-20 | Captive | Red rabbit |
| 91D046 | Sink | Feces | Dayi | Oct-20 | Captive | Red rabbit |
| 01D086 | Sink | Feces | Dayi | Oct-20 | Captive | Red rabbit |
| 01D031 | Sink | Feces | Dayi | Oct-20 | Captive | Red rabbit |
| 95D104 | Sink | Feces | Dayi | Oct-20 | Captive | Red rabbit |
| 92D149 | Sink | Feces | Dayi | Oct-20 | Captive | Red rabbit |
| 91D021 | Sink | Feces | Dayi | Oct-20 | Captive | Red rabbit |
| 01D338 | Sink | Feces | Dayi | Oct-20 | Captive | Red rabbit |
| FC | Source | Diet | Dayi | Oct-20 | Captive | Forage |
| FT | Source | Diet | Dayi | Oct-20 | Captive | Forage |
| CF1 | Source | Diet | Dujiangyan | Nov-19 | Captive | Forage |
| EL1 | Source | Diet | Dujiangyan | Nov-19 | Captive | Leaves of *Ulmus pumila* L. |
| EL2 | Source | Diet | Dujiangyan | Nov-19 | Captive | Leaves of *Ulmus pumila* L. |
| EL3 | Source | Diet | Dujiangyan | Nov-19 | Captive | Leaves of *Ulmus pumila* L. |
| EL4 | Source | Diet | Dujiangyan | Nov-19 | Captive | Leaves of *Ulmus pumila* L. |
| EL5 | Source | Diet | Dujiangyan | Nov-19 | Captive | Leaves of *Ulmus pumila* L. |
| BL1 | Source | Diet | Dujiangyan | Nov-19 | Captive | *Melia azedarach* |
| BL2 | Source | Diet | Dujiangyan | Nov-19 | Captive | *Melia azedarach* |
| BL3 | Source | Diet | Dujiangyan | Nov-19 | Captive | *Melia azedarach* |
| BL4 | Source | Diet | Dujiangyan | Nov-19 | Captive | *Melia azedarach* |
| BL5 | Source | Diet | Dujiangyan | Nov-19 | Captive | *Melia azedarach* |
| TM1 | Source | Diet | Dujiangyan | Nov-19 | Captive | *Acer buergerianum* Miq. |
| TM2 | Source | Diet | Dujiangyan | Nov-19 | Captive | *Acer buergerianum* Miq. |
| TM3 | Source | Diet | Dujiangyan | Nov-19 | Captive | *Acer buergerianum* Miq. |
| TM4 | Source | Diet | Dujiangyan | Nov-19 | Captive | *Acer buergerianum* Miq. |
| TM5 | Source | Diet | Dujiangyan | Nov-19 | Captive | *Acer buergerianum* Miq. |
| FM1 | Source | Diet | Dujiangyan | Nov-19 | Captive | *Morus alba* Linn. Sp. |
| FM2 | Source | Diet | Dujiangyan | Nov-19 | Captive | *Morus alba* Linn. Sp. |
| FM3 | Source | Diet | Dujiangyan | Nov-19 | Captive | *Morus alba* Linn. Sp. |
| FM4 | Source | Diet | Dujiangyan | Nov-19 | Captive | *Morus alba* Linn. Sp. |
| FM5 | Source | Diet | Dujiangyan | Nov-19 | Captive | *Morus alba* Linn. Sp. |
| GC1 | Source | Diet | Dujiangyan | Nov-19 | Captive | *Capparis sepiaria* L. |
| GC2 | Source | Diet | Dujiangyan | Nov-19 | Captive | *Capparis sepiaria* L. |
| GC3 | Source | Diet | Dujiangyan | Nov-19 | Captive | *Capparis sepiaria* L. |
| GC4 | Source | Diet | Dujiangyan | Nov-19 | Captive | *Capparis sepiaria* L. |
| GC5 | Source | Diet | Dujiangyan | Nov-19 | Captive | *Capparis sepiaria* L. |
| GD1 | Source | Diet | Dujiangyan | Nov-19 | Captive | Forage |
| BM1 | Source | Diet | Dujiangyan | Nov-19 | Captive | Forage |
| DJY57G | Sink | Feces | Dujiangyan | Nov-19 | Captive | Musk deer (*Moschus moschiferus*) |
| DJY58G | Sink | Feces | Dujiangyan | Nov-19 | Captive | Musk deer (*Moschus moschiferus*) |
| DJY59G | Sink | Feces | Dujiangyan | Nov-19 | Captive | Musk deer (*Moschus moschiferus*) |
| DJY60G | Sink | Feces | Dujiangyan | Nov-19 | Captive | Musk deer (*Moschus moschiferus*) |
| DJY61G | Sink | Feces | Dujiangyan | Nov-19 | Captive | Musk deer (*Moschus moschiferus*) |
| DJY62G | Sink | Feces | Dujiangyan | Nov-19 | Captive | Musk deer (*Moschus moschiferus*) |
| DJY63G | Sink | Feces | Dujiangyan | Nov-19 | Captive | Musk deer (*Moschus moschiferus*) |
| DJY64G | Sink | Feces | Dujiangyan | Nov-19 | Captive | Musk deer (*Moschus moschiferus*) |
| DJY65G | Sink | Feces | Dujiangyan | Nov-19 | Captive | Musk deer (*Moschus moschiferus*) |
| DJY66G | Sink | Feces | Dujiangyan | Nov-19 | Captive | Musk deer (*Moschus moschiferus*) |
| DJY67G | Sink | Feces | Dujiangyan | Nov-19 | Captive | Musk deer (*Moschus moschiferus*) |
| DJY68G | Sink | Feces | Dujiangyan | Nov-19 | Captive | Musk deer (*Moschus moschiferus*) |
| DJY69G | Sink | Feces | Dujiangyan | Nov-19 | Captive | Musk deer (*Moschus moschiferus*) |
| DJY70G | Sink | Feces | Dujiangyan | Nov-19 | Captive | Musk deer (*Moschus moschiferus*) |
| DJY71G | Sink | Feces | Dujiangyan | Nov-19 | Captive | Musk deer (*Moschus moschiferus*) |
| DJY72G | Sink | Feces | Dujiangyan | Nov-19 | Captive | Musk deer (*Moschus moschiferus*) |
| DJY73G | Sink | Feces | Dujiangyan | Nov-19 | Captive | Musk deer (*Moschus moschiferus*) |
| DJY74G | Sink | Feces | Dujiangyan | Nov-19 | Captive | Musk deer (*Moschus moschiferus*) |
| DJY75G | Sink | Feces | Dujiangyan | Nov-19 | Captive | Musk deer (*Moschus moschiferus*) |
| DJY76G | Sink | Feces | Dujiangyan | Nov-19 | Captive | Musk deer (*Moschus moschiferus*) |
| DJY77G | Sink | Feces | Dujiangyan | Nov-19 | Captive | Musk deer (*Moschus moschiferus*) |
| DJY78G | Sink | Feces | Dujiangyan | Nov-19 | Captive | Musk deer (*Moschus moschiferus*) |
| DJY79G | Sink | Feces | Dujiangyan | Nov-19 | Captive | Musk deer (*Moschus moschiferus*) |
| DJY80G | Sink | Feces | Dujiangyan | Nov-19 | Captive | Musk deer (*Moschus moschiferus*) |
| DJY81G | Sink | Feces | Dujiangyan | Nov-19 | Captive | Musk deer (*Moschus moschiferus*) |
| DJY82G | Sink | Feces | Dujiangyan | Nov-19 | Captive | Musk deer (*Moschus moschiferus*) |
| DJY83G | Sink | Feces | Dujiangyan | Nov-19 | Captive | Musk deer (*Moschus moschiferus*) |
| DJY84G | Sink | Feces | Dujiangyan | Nov-19 | Captive | Musk deer (*Moschus moschiferus*) |
| DJY85G | Sink | Feces | Dujiangyan | Nov-19 | Captive | Musk deer (*Moschus moschiferus*) |
| DJY86G | Sink | Feces | Dujiangyan | Nov-19 | Captive | Musk deer (*Moschus moschiferus*) |
| DJY87G | Sink | Feces | Dujiangyan | Nov-19 | Captive | Musk deer (*Moschus moschiferus*) |
| DJY88G | Sink | Feces | Dujiangyan | Nov-19 | Captive | Musk deer (*Moschus moschiferus*) |
| DJY89G | Sink | Feces | Dujiangyan | Nov-19 | Captive | Musk deer (*Moschus moschiferus*) |
| DJY90G | Sink | Feces | Dujiangyan | Nov-19 | Captive | Musk deer (*Moschus moschiferus*) |
| DJY91G | Sink | Feces | Dujiangyan | Nov-19 | Captive | Musk deer (*Moschus moschiferus*) |
| DJY92G | Sink | Feces | Dujiangyan | Nov-19 | Captive | Musk deer (*Moschus moschiferus*) |
| DJY93G | Sink | Feces | Dujiangyan | Nov-19 | Captive | Musk deer (*Moschus moschiferus*) |
| DJY94G | Sink | Feces | Dujiangyan | Nov-19 | Captive | Musk deer (*Moschus moschiferus*) |
| DJY95G | Sink | Feces | Dujiangyan | Nov-19 | Captive | Musk deer (*Moschus moschiferus*) |
| DJY96G | Sink | Feces | Dujiangyan | Nov-19 | Captive | Musk deer (*Moschus moschiferus*) |
| DJY97G | Sink | Feces | Dujiangyan | Nov-19 | Captive | Musk deer (*Moschus moschiferus*) |
| DJY98G | Sink | Feces | Dujiangyan | Nov-19 | Captive | Musk deer (*Moschus moschiferus*) |
| DJY99G | Sink | Feces | Dujiangyan | Nov-19 | Captive | Musk deer (*Moschus moschiferus*) |
| DJY100G | Sink | Feces | Dujiangyan | Nov-19 | Captive | Musk deer (*Moschus moschiferus*) |
| 231 | Source | Diet | Dafeng | Jun-17 | Wild | *Pennisetum purpureum* Schumach |
| 252 | Source | Diet | Dafeng | Jun-17 | Wild | *Pennisetum purpureum* Schumach |
| 342LWC-1 | Source | Diet | Dafeng | Sep-17 | Wild | *Pennisetum purpureum* Schumach |
| 342LWC-2 | Source | Diet | Dafeng | Sep-17 | Wild | *Pennisetum purpureum* Schumach |
| 342LWC-3 | Source | Diet | Dafeng | Sep-17 | Wild | *Imperata cylindrica* |
| 924 | Source | Diet | Dafeng | May-17 | Wild | *Imperata cylindrica* |
| 219 | Source | Diet | Dafeng | Jun-17 | Wild | *Spartina alterniflora* |
| 917-1 | Source | Diet | Dafeng | May-17 | Wild | *Spartina alterniflora* |
| 917-2 | Source | Diet | Dafeng | May-17 | Wild | *Spartina alterniflora* |
| 216-10 | Source | Diet | Dafeng | Jun-17 | Wild | *Spartina alterniflora* |
| 216-3 | Source | Diet | Dafeng | Jun-17 | Wild | *Spartina alterniflora* |
| 216-4 | Source | Diet | Dafeng | Jun-17 | Wild | *Spartina alterniflora* |
| 216-9 | Source | Diet | Dafeng | Jun-17 | Wild | *Spartina alterniflora* |
| 350HHMC-1 | Source | Diet | Dafeng | Sep-17 | Wild | *Spartina alterniflora* |
| 353HHMC-1 | Source | Diet | Dafeng | Sep-17 | Wild | *Spartina alterniflora* |
| 353HHMC-2 | Source | Diet | Dafeng | Sep-17 | Wild | *Spartina alterniflora* |
| 357HHMC-1 | Source | Diet | Dafeng | Sep-17 | Wild | *Spartina alterniflora* |
| 357HHMC-2 | Source | Diet | Dafeng | Sep-17 | Wild | *Spartina alterniflora* |
| 925-2 | Source | Diet | Dafeng | May-17 | Wild | *Phragmites australis* |
| 925-3 | Source | Diet | Dafeng | May-17 | Wild | *Phragmites australis* |
| 179-1 | Source | Diet | Dafeng | Jun-17 | Wild | *Phragmites australis* |
| 179-2 | Source | Diet | Dafeng | Jun-17 | Wild | *Phragmites australis* |
| 208-2 | Source | Diet | Dafeng | Jun-17 | Wild | *Phragmites australis* |
| 208-3 | Source | Diet | Dafeng | Jun-17 | Wild | *Phragmites australis* |
| 341LW-1 | Source | Diet | Dafeng | Sep-17 | Wild | *Phragmites australis* |
| 341LW-2 | Source | Diet | Dafeng | Sep-17 | Wild | *Phragmites australis* |
| 351LW-1 | Source | Diet | Dafeng | Sep-17 | Wild | *Phragmites australis* |
| 351LW-2 | Source | Diet | Dafeng | Sep-17 | Wild | *Phragmites australis* |
| 351song-1 | Source | Diet | Dafeng | Sep-17 | Wild | *Phragmites australis* |
| 910 | Source | Diet | Dafeng | May-17 | Wild | *Suaeda glauca* |
| 215 | Source | Diet | Dafeng | Jun-17 | Wild | *Suaeda glauca* |
| 345JP-1 | Source | Diet | Dafeng | Sep-17 | Wild | *Suaeda glauca* |
| 345JP-2 | Source | Diet | Dafeng | Sep-17 | Wild | *Suaeda glauca* |
| 347JP-1 | Source | Diet | Dafeng | Sep-17 | Wild | *Suaeda glauca* |
| 338-1 | Sink | Feces | Dafeng | Jun-17 | Wild | Locust |
| 338-3 | Sink | Feces | Dafeng | Jun-17 | Wild | Locust |
| 339-1 | Sink | Feces | Dafeng | Jun-17 | Wild | Locust |
| 339-2 | Sink | Feces | Dafeng | Jun-17 | Wild | Locust |
| 339-3 | Sink | Feces | Dafeng | Jun-17 | Wild | Locust |
| DFHC351-3 | Sink | Feces | Dafeng | Aug-17 | Wild | Locust |
| DFHC349-2 | Sink | Feces | Dafeng | Aug-17 | Wild | Locust |
| DFHC349-3 | Sink | Feces | Dafeng | Aug-17 | Wild | Locust |
| DFHC353-1 | Sink | Feces | Dafeng | Aug-17 | Wild | Locust |
| DFHC353-2 | Sink | Feces | Dafeng | Aug-17 | Wild | Locust |
| DFHC353-3 | Sink | Feces | Dafeng | Jun-17 | Wild | Locust |
| 178-1 | Sink | Feces | Dafeng | Jun-17 | Wild | Locust |
| 334-1 | Sink | Feces | Dafeng | Jun-17 | Wild | Locust |
| 334-2 | Sink | Feces | Dafeng | Jun-17 | Wild | Locust |
| 334-3 | Sink | Feces | Dafeng | Jun-17 | Wild | Locust |
| 335-1 | Sink | Feces | Dafeng | Jun-17 | Wild | Locust |
| 335-2 | Sink | Feces | Dafeng | Jun-17 | Wild | Locust |
| 335-3 | Sink | Feces | Dafeng | Jun-17 | Wild | Locust |
| 336-1 | Sink | Feces | Dafeng | Jun-17 | Wild | Locust |
| 336-3 | Sink | Feces | Dafeng | Jun-17 | Wild | Locust |
| 178-2 | Sink | Feces | Dafeng | Jul-17 | Wild | Locust |
| 344-song1 | Sink | Feces | Dafeng | Sep-17 | Wild | Locust |
| 338-ZS | Sink | Feces | Dafeng | Jun-17 | Wild | Tettigoniidae |
| 339-ZS | Sink | Feces | Dafeng | Jun-17 | Wild | Tettigoniidae |
| 928-2 | Sink | Feces | Dafeng | May-17 | Wild | Tettigoniidae |
| 335-1ZS | Sink | Feces | Dafeng | Jun-17 | Wild | Tettigoniidae |
| 335-2ZS | Sink | Feces | Dafeng | Jun-17 | Wild | Tettigoniidae |
| 221-1 | Sink | Feces | Dafeng | Jun-17 | Wild | Père David's Deer (*Elaphurus davidianus*, Milu) |
| 221-2 | Sink | Feces | Dafeng | Jun-17 | Wild | Père David's Deer (*Elaphurus davidianus*, Milu) |
| 221-3 | Sink | Feces | Dafeng | Jun-17 | Wild | Père David's Deer (*Elaphurus davidianus*, Milu) |
| 221-4 | Sink | Feces | Dafeng | Jun-17 | Wild | Père David's Deer (*Elaphurus davidianus*, Milu) |
| 221-5 | Sink | Feces | Dafeng | Jun-17 | Wild | Père David's Deer (*Elaphurus davidianus*, Milu) |
| 221-6 | Sink | Feces | Dafeng | Jun-17 | Wild | Père David's Deer (*Elaphurus davidianus*, Milu) |
| 221-7 | Sink | Feces | Dafeng | Jun-17 | Wild | Père David's Deer (*Elaphurus davidianus*, Milu) |
| 221-8 | Sink | Feces | Dafeng | Jun-17 | Wild | Père David's Deer (*Elaphurus davidianus*, Milu) |
| 222-1 | Sink | Feces | Dafeng | Jun-17 | Wild | Père David's Deer (*Elaphurus davidianus*, Milu) |
| 222-2 | Sink | Feces | Dafeng | Jun-17 | Wild | Père David's Deer (*Elaphurus davidianus*, Milu) |
| 222-3 | Sink | Feces | Dafeng | Jun-17 | Wild | Père David's Deer (*Elaphurus davidianus*, Milu) |
| 223-1 | Sink | Feces | Dafeng | Jun-17 | Wild | Père David's Deer (*Elaphurus davidianus*, Milu) |
| 223-2 | Sink | Feces | Dafeng | Jun-17 | Wild | Père David's Deer (*Elaphurus davidianus*, Milu) |
| 223-3 | Sink | Feces | Dafeng | Jun-17 | Wild | Père David's Deer (*Elaphurus davidianus*, Milu) |
| 223-4 | Sink | Feces | Dafeng | Jun-17 | Wild | Père David's Deer (*Elaphurus davidianus*, Milu) |
| 346-ML-1 | Sink | Feces | Dafeng | Sep-17 | Wild | Père David's Deer (*Elaphurus davidianus*, Milu) |
| 346-ML-2 | Sink | Feces | Dafeng | Sep-17 | Wild | Père David's Deer (*Elaphurus davidianus*, Milu) |
| 346-ML-3 | Sink | Feces | Dafeng | Sep-17 | Wild | Père David's Deer (*Elaphurus davidianus*, Milu) |
| 346-ML-4 | Sink | Feces | Dafeng | Sep-17 | Wild | Père David's Deer (*Elaphurus davidianus*, Milu) |
| 346-ML-5 | Sink | Feces | Dafeng | Sep-17 | Wild | Père David's Deer (*Elaphurus davidianus*, Milu) |
| 354ML-1 | Sink | Feces | Dafeng | Sep-17 | Wild | Père David's Deer (*Elaphurus davidianus*, Milu) |
| 354ML-2 | Sink | Feces | Dafeng | Sep-17 | Wild | Père David's Deer (*Elaphurus davidianus*, Milu) |
| 354ML-3 | Sink | Feces | Dafeng | Sep-17 | Wild | Père David's Deer (*Elaphurus davidianus*, Milu) |
| 354ML-4 | Sink | Feces | Dafeng | Sep-17 | Wild | Père David's Deer (*Elaphurus davidianus*, Milu) |
| 354ML-5 | Sink | Feces | Dafeng | Sep-17 | Wild | Père David's Deer (*Elaphurus davidianus*, Milu) |
| 356ML-1 | Sink | Feces | Dafeng | Sep-17 | Wild | Père David's Deer (*Elaphurus davidianus*, Milu) |
| 356ML-2 | Sink | Feces | Dafeng | Sep-17 | Wild | Père David's Deer (*Elaphurus davidianus*, Milu) |
| 356ML-3 | Sink | Feces | Dafeng | Sep-17 | Wild | Père David's Deer (*Elaphurus davidianus*, Milu) |
| 356ML-4 | Sink | Feces | Dafeng | Sep-17 | Wild | Père David's Deer (*Elaphurus davidianus*, Milu) |
| 356ML-5 | Sink | Feces | Dafeng | Sep-17 | Wild | Père David's Deer (*Elaphurus davidianus*, Milu) |
| 907-1 | Sink | Feces | Dafeng | May-17 | Wild | Père David's Deer (*Elaphurus davidianus*, Milu) |
| 907-2 | Sink | Feces | Dafeng | May-17 | Wild | Père David's Deer (*Elaphurus davidianus*, Milu) |
| 907-3 | Sink | Feces | Dafeng | May-17 | Wild | Père David's Deer (*Elaphurus davidianus*, Milu) |
| 907-4 | Sink | Feces | Dafeng | May-17 | Wild | Père David's Deer (*Elaphurus davidianus*, Milu) |
| 909-1 | Sink | Feces | Dafeng | May-17 | Wild | Père David's Deer (*Elaphurus davidianus*, Milu) |
| 909-2 | Sink | Feces | Dafeng | May-17 | Wild | Père David's Deer (*Elaphurus davidianus*, Milu) |
| 909-3 | Sink | Feces | Dafeng | May-17 | Wild | Père David's Deer (*Elaphurus davidianus*, Milu) |
| 914-1 | Sink | Feces | Dafeng | May-17 | Wild | Père David's Deer (*Elaphurus davidianus*, Milu) |
| 915-3 | Sink | Feces | Dafeng | May-17 | Wild | Père David's Deer (*Elaphurus davidianus*, Milu) |
| 918-1 | Sink | Feces | Dafeng | May-17 | Wild | Père David's Deer (*Elaphurus davidianus*, Milu) |
| 918-2 | Sink | Feces | Dafeng | May-17 | Wild | Père David's Deer (*Elaphurus davidianus*, Milu) |
| 918-3 | Sink | Feces | Dafeng | May-17 | Wild | Père David's Deer (*Elaphurus davidianus*, Milu) |
| 919-2 | Sink | Feces | Dafeng | May-17 | Wild | Père David's Deer (*Elaphurus davidianus*, Milu) |
| 923-1 | Sink | Feces | Dafeng | May-17 | Wild | Père David's Deer (*Elaphurus davidianus*, Milu) |
| 923-2 | Sink | Feces | Dafeng | May-17 | Wild | Père David's Deer (*Elaphurus davidianus*, Milu) |
| lycc1 | Sink | Gut contents | Liyang | Jul-18 | Wild | Cicadidae adult |
| lycc2 | Sink | Gut contents | Liyang | Jul-18 | Wild | Cicadidae adult |
| lycc3 | Sink | Gut contents | Liyang | Jul-18 | Wild | Cicadidae adult |
| lycc4 | Sink | Gut contents | Liyang | Jul-18 | Wild | Cicadidae adult |
| lycc5 | Sink | Gut contents | Liyang | Jul-18 | Wild | Cicadidae adult |
| lycc6 | Sink | Gut contents | Liyang | Jul-18 | Wild | Cicadidae adult |
| 154-NCC1 | Sink | Gut contents | Liyang | Aug-17 | Wild | Cicadidae adult |
| 154-NCC2 | Sink | Gut contents | Liyang | Aug-17 | Wild | Cicadidae adult |
| 154-NCC4 | Sink | Gut contents | Liyang | Aug-17 | Wild | Cicadidae adult |
| 154-NCC5 | Sink | Gut contents | Liyang | Aug-17 | Wild | Cicadidae adult |
| 154-NCC8 | Sink | Gut contents | Liyang | Aug-17 | Wild | Cicadidae adult |
| 154-NCC9 | Sink | Gut contents | Liyang | Aug-17 | Wild | Cicadidae adult |
| 154NCC10 | Sink | Gut contents | Liyang | Aug-17 | Wild | Cicadidae adult |
| 154NCC3 | Sink | Gut contents | Liyang | Aug-17 | Wild | Cicadidae adult |
| 154NCC6 | Sink | Gut contents | Liyang | Aug-17 | Wild | Cicadidae adult |
| 154NCC7 | Sink | Gut contents | Liyang | Aug-17 | Wild | Cicadidae adult |
| 18YXMZ | Source | Diet | Liyang | Jul-18 | Wild | *Phyllostachys heterocycla* (Carr.) Mitford cv. Pubescens |
| 153-NZ1 | Source | Diet | Liyang | Aug-17 | Wild | *Phyllostachys heterocycla* (Carr.) Mitford cv. Pubescens |
| 153-NZ2 | Source | Diet | Liyang | Aug-17 | Wild | *Phyllostachys heterocycla* (Carr.) Mitford cv. Pubescens |
| 154-NZ1 | Source | Diet | Liyang | Aug-17 | Wild | *Phyllostachys heterocycla* (Carr.) Mitford cv. Pubescens |
| 154-NZ2 | Source | Diet | Liyang | Aug-17 | Wild | *Phyllostachys heterocycla* (Carr.) Mitford cv. Pubescens |
| 153-NZM3 | Sink | Gut contents | Liyang | Aug-17 | Wild | Pyralidae |
| 153-NZM4 | Sink | Gut contents | Liyang | Aug-17 | Wild | Pyralidae |
| 153-NZM5 | Sink | Gut contents | Liyang | Aug-17 | Wild | Pyralidae |
| 153-NZM6 | Sink | Gut contents | Liyang | Aug-17 | Wild | Pyralidae |
| 153-NZM1 | Sink | Gut contents | Liyang | Aug-17 | Wild | Pyralidae |
| 153-NZM2 | Sink | Gut contents | Liyang | Aug-17 | Wild | Pyralidae |
| xzccy1 | Sink | Gut contents | Xuzhou | Jun-18 | Wild | Cicadidae adult |
| xzccy2 | Sink | Gut contents | Xuzhou | Jun-18 | Wild | Cicadidae adult |
| xzccy3 | Sink | Gut contents | Xuzhou | Jun-18 | Wild | Cicadidae adult |
| xzccy4 | Sink | Gut contents | Xuzhou | Jun-18 | Wild | Cicadidae adult |
| xzccy5 | Sink | Gut contents | Xuzhou | Jun-18 | Wild | Cicadidae adult |
| XCC1 | Sink | Gut contents | Xuzhou | Aug-17 | Wild | Cicadidae adult |
| XCC10 | Sink | Gut contents | Xuzhou | Aug-17 | Wild | Cicadidae adult |
| XCC5 | Sink | Gut contents | Xuzhou | Aug-17 | Wild | Cicadidae adult |
| XCC7 | Sink | Gut contents | Xuzhou | Aug-17 | Wild | Cicadidae adult |
| XCC8 | Sink | Gut contents | Xuzhou | Aug-17 | Wild | Cicadidae adult |
| XCC9 | Sink | Gut contents | Xuzhou | Aug-17 | Wild | Cicadidae adult |
| xzccl1 | Sink | Gut contents | Xuzhou | Jun-18 | Wild | Cicadidae adult |
| xzccl2 | Sink | Gut contents | Xuzhou | Jun-18 | Wild | Cicadidae adult |
| xzccl3 | Sink | Gut contents | Xuzhou | Jun-18 | Wild | Cicadidae adult |
| xzccl4 | Sink | Gut contents | Xuzhou | Jun-18 | Wild | Cicadidae adult |
| xzccl5 | Sink | Gut contents | Xuzhou | Jun-18 | Wild | Cicadidae adult |
| xzccl6 | Sink | Gut contents | Xuzhou | Jun-18 | Wild | Cicadidae adult |
| xzcch2 | Sink | Gut contents | Xuzhou | Jun-18 | Wild | Cicadidae adult |
| xzcch3 | Sink | Gut contents | Xuzhou | Jun-18 | Wild | Cicadidae adult |
| xzcch4 | Sink | Gut contents | Xuzhou | Jun-18 | Wild | Cicadidae adult |
| xzcch5 | Sink | Gut contents | Xuzhou | Jun-18 | Wild | Cicadidae adult |
| xzcch6 | Sink | Gut contents | Xuzhou | Jun-18 | Wild | Cicadidae adult |
| 18XZYS | Source | Diet | Xuzhou | Aug-17 | Wild | *Populus* L. |
| yangshugen360 | Source | Diet | Xuzhou | Aug-17 | Wild | *Populus* L. |
| yangshugen361 | Source | Diet | Xuzhou | Aug-17 | Wild | *Populus* L. |
| yangshu360-1 | Source | Diet | Xuzhou | Aug-17 | Wild | *Populus* L. |
| yangshu360-2 | Source | Diet | Xuzhou | Aug-17 | Wild | *Populus* L. |
| yangshujingye361 | Source | Diet | Xuzhou | Aug-17 | Wild | *Populus* L. |
| 237-ZJAJ1 | Source | Diet | Anji | Aug-17 | Wild | *Phyllostachys platyglossa* |
| 237-ZJAJ2 | Source | Diet | Anji | Aug-17 | Wild | *Phyllostachys platyglossa* |
| 236-ZJAJ1 | Source | Diet | Anji | Aug-17 | Wild | *Fargesia semicoriacea* Yi |
| 236-ZJAJ2 | Source | Diet | Anji | Aug-17 | Wild | *Fargesia semicoriacea* Yi |
| 235-ZJAJ1 | Source | Diet | Anji | Aug-17 | Wild | *Phyllostachys heterocycla* (Carr.) Mitford cv. Pubescens |
| 235-ZJAJ2 | Source | Diet | Anji | Aug-17 | Wild | *Phyllostachys heterocycla* (Carr.) Mitford cv. Pubescens |
| 231-ZJAJ1 | Source | Diet | Anji | Aug-17 | Wild | *Phyllostachys heterocycla* (Carr.) Mitford cv. Pubescens |
| 231-ZJAJ2 | Source | Diet | Anji | Aug-17 | Wild | *Phyllostachys heterocycla* (Carr.) Mitford cv. Pubescens |
| 232-ZJAJ | Source | Diet | Anji | Aug-17 | Wild | *Phyllostachys heterocycla* (Carr.) Mitford cv. Pubescens |
| 233-ZJAJ | Source | Diet | Anji | Aug-17 | Wild | *Phyllostachys heterocycla* (Carr.) Mitford cv. Pubescens |
| 234-ZJAJ | Source | Diet | Anji | Aug-17 | Wild | *Phyllostachys heterocycla* (Carr.) Mitford cv. Pubescens |
| 231-ZJZM2 | Sink | Gut contents | Anji | Aug-17 | Wild | Pyralidae |
| 231-ZJZM3 | Sink | Gut contents | Anji | Aug-17 | Wild | Pyralidae |
| 231-ZJZM4 | Sink | Gut contents | Anji | Aug-17 | Wild | Pyralidae |
| 231-ZJZN1 | Sink | Gut contents | Anji | Aug-17 | Wild | Pyralidae |
| 231ZJZM5 | Sink | Gut contents | Anji | Aug-17 | Wild | Pyralidae |
| 232-ZJZM1 | Sink | Gut contents | Anji | Aug-17 | Wild | Pyralidae |
| 232-ZJZM2 | Sink | Gut contents | Anji | Aug-17 | Wild | Pyralidae |
| 233-ZJZM1 | Sink | Gut contents | Anji | Aug-17 | Wild | Pyralidae |
| 233-ZJZM2 | Sink | Gut contents | Anji | Aug-17 | Wild | Pyralidae |
| 233-ZJZM3 | Sink | Gut contents | Anji | Aug-17 | Wild | Pyralidae |

Table S2 The shared ASVs in the microbiome only between the herbivorous insects and dietary plants in this study

| ASV ID | Taxonomy |
| --- | --- |
| 6f158a22df821e35ff1311aeb52563d0 | d__Bacteria; p__Proteobacteria; c__Gammaproteobacteria; o__Enterobacteriales; f__Enterobacteriaceae; g__Pantoea |
| 89a990723fa4f138ded56583fe0578d6 | d__Bacteria; p__Proteobacteria; c__Gammaproteobacteria; o__Enterobacteriales; f__Enterobacteriaceae; g__Escherichia-Shigella |
| db763fd81e8bbffe8d937b0b8e34ef3c | d__Bacteria; p__Firmicutes; c__Bacilli; o__Bacillales; f__Staphylococcaceae; g__Staphylococcus |
| 924a0df32183337f419838da01fe9552 | d__Bacteria; p__Proteobacteria; c__Gammaproteobacteria; o__Enterobacteriales; f__Enterobacteriaceae; g__Enterobacter |
| 2ec54d54feb48741aa720905db5323c8 | d__Bacteria; p__Firmicutes; c__Bacilli; o__Bacillales; f__Staphylococcaceae; g__Staphylococcus |
| 175102838006ec45bcc93faeca409148 | d__Bacteria; p__Proteobacteria; c__Gammaproteobacteria; o__Enterobacteriales; f__Enterobacteriaceae; g__Pantoea |
| 4c9dd3b3429079efca968330ca1d4f95 | d__Bacteria; p__Proteobacteria; c__Gammaproteobacteria; o__Enterobacteriales; f__Enterobacteriaceae; g__Klebsiella; s__uncultured bacterium |
| 41a6f7b85a41337d025d9a3a0c5a4314 | d__Bacteria; p__Proteobacteria; c__Gammaproteobacteria; o__Oceanospirillales; f__Halomonadaceae; g__Salinicola |
| c5aec1c42911fac4dae9ffef1054c4f6 | d__Bacteria; p__Proteobacteria; c__Alphaproteobacteria |
| ba9d344240cbb61710b3d880d3e15ea5 | d__Bacteria; p__Proteobacteria; c__Gammaproteobacteria; o__Enterobacteriales; f__Enterobacteriaceae; g__Kluyvera; s__Kluyvera ascorbata |
| bc9d6ceaca20f6915baee07c422992b1 | d__Bacteria; p__Firmicutes; c__Bacilli; o__Lactobacillales; f__Streptococcaceae; g__Lactococcus; s__Lactococcus garvieae ATCC 49156 |
| 378bbe3314e043127a3270b7aa29b361 | d__Bacteria; p__Proteobacteria; c__Gammaproteobacteria; o__Enterobacteriales; f__Enterobacteriaceae; g__Pantoea |
| f20920ccc20ad14898f6d3dc6df54d94 | d__Bacteria; p__Firmicutes; c__Bacilli; o__Bacillales; f__Family XII; g__Exiguobacterium |
| a2586f9b345e6746a32b8c008cfac86c | d__Bacteria; p__Proteobacteria; c__Gammaproteobacteria; o__Xanthomonadales; f__Xanthomonadaceae; g__Stenotrophomonas; s__uncultured bacterium |
| 7d9a4b1ae1052575516e07604d9669c6 | d__Bacteria; p__Proteobacteria; c__Gammaproteobacteria; o__Vibrionales; f__Vibrionaceae; g__Vibrio; s__Vibrio fluvialis |
| 866cecbf8c88dfb66e0f6d48d8caf59a | d__Bacteria; p__Firmicutes; c__Bacilli; o__Bacillales; f__Bacillaceae; g__Bacillus |
| 96936a2da927f5dc76dae0c74974cf1c | d__Bacteria; p__Proteobacteria; c__Gammaproteobacteria; o__Oceanospirillales; f__Halomonadaceae; g__Salinicola |
| 5a945c70cef87b24270a4d0800dca85f | d__Bacteria; p__Proteobacteria; c__Gammaproteobacteria; o__Enterobacteriales; f__Enterobacteriaceae; g__Citrobacter |
| 848cf63cb712ec87e5026648c689b0ae | d__Bacteria; p__Proteobacteria; c__Gammaproteobacteria; o__Enterobacteriales; f__Enterobacteriaceae; g__Pantoea |
| d2f369649a8fd32da2078c5fe576ccaa | d__Bacteria; p__Actinobacteria; c__Actinobacteria; o__Micrococcales; f__Microbacteriaceae; g__Curtobacterium |
| 236b3720998174bdeefab2ec777aab8f | d__Bacteria; p__Firmicutes; c__Bacilli; o__Bacillales; f__Bacillaceae; g__Bacillus; s__Bacillus cereus |
| 183d6e320cdb9c7e395405ca390cbb6c | d__Bacteria; p__Proteobacteria; c__Gammaproteobacteria; o__Pseudomonadales; f__Moraxellaceae; g__Acinetobacter |
| 4b4a9477c7e9cdde9bdfae18ffc7aaee | d__Bacteria; p__Proteobacteria; c__Gammaproteobacteria; o__Alteromonadales; f__Shewanellaceae; g__Shewanella |
| f7edee413ae42becd1005ce369dee114 | d__Bacteria; p__Firmicutes; c__Bacilli; o__Bacillales; f__Bacillaceae; g__Bacillus; s__Bacillus megaterium |
| 72dbeb655eb445f89e013ef92e28ee5e | d__Bacteria; p__Firmicutes; c__Bacilli; o__Lactobacillales; f__Enterococcaceae; g__Enterococcus |
| 9747fb1869f3cd864afc289a6828200a | d__Bacteria; p__Proteobacteria; c__Gammaproteobacteria; o__Vibrionales; f__Vibrionaceae; g__Vibrio; s__Vibrio porteresiae |
| 869f2f98bfd3afe245251a1c2a1755a7 | d__Bacteria; p__Proteobacteria; c__Gammaproteobacteria; o__Xanthomonadales; f__Xanthomonadaceae; g__Stenotrophomonas; s__uncultured bacterium |
| 36c2b2f944e22bdc3c54ce8af1ad2d59 | d__Bacteria; p__Proteobacteria; c__Gammaproteobacteria; o__Vibrionales; f__Vibrionaceae; g__Vibrio |
| b68d64ad6b05f9f471fb92f895e46e93 | d__Bacteria; p__Firmicutes; c__Bacilli; o__Bacillales; f__Family XII; g__Exiguobacterium; s__Exiguobacterium indicum |
| 1a5f1a0c6d808853cdeb956bb49ce6bb | d__Bacteria; p__Proteobacteria; c__Alphaproteobacteria; o__Sphingomonadales; f__Sphingomonadaceae; g__Stakelama; s__Stakelama pacifica |
| a2a2b3ceb8e018b8d484c0515cf5cd67 | d__Bacteria; p__Proteobacteria; c__Gammaproteobacteria; o__Oceanospirillales; f__Halomonadaceae; g__Kushneria; s__Kushneria indalinina |
| d19aa79967d4554fd9e655bf829ee022 | d__Bacteria; p__Actinobacteria; c__Actinobacteria; o__Micrococcales; f__Microbacteriaceae; g__Curtobacterium |
| 40ce13c089794bce8db33b8a1add907d | d__Bacteria; p__Proteobacteria; c__Gammaproteobacteria; o__Xanthomonadales; f__Xanthomonadaceae; g__Pseudoxanthomonas; s__uncultured bacterium |
| 81d9c09d74a6cb51fd4afa31b871ac41 | d__Bacteria; p__Bacteroidetes; c__Sphingobacteriia; o__Sphingobacteriales; f__Sphingobacteriaceae; g__Sphingobacterium |
| 3a13e006cd5aa43b469e939778e66331 | d__Bacteria; p__Proteobacteria; c__Gammaproteobacteria; o__Enterobacteriales; f__Enterobacteriaceae; g__Citrobacter; s__uncultured bacterium |
| a7de04c5a61a05b863c8456b927d3bb6 | d__Bacteria; p__Proteobacteria; c__Gammaproteobacteria; o__Enterobacteriales; f__Enterobacteriaceae; g__Klebsiella |
| bd629defa829deb7651cc12d0bc092af | d__Bacteria; p__Firmicutes; c__Bacilli; o__Bacillales; f__Planococcaceae; g__Planomicrobium |
| 84349eff10ac381585a0db6eb3fc6ccf | d__Bacteria; p__Proteobacteria; c__Gammaproteobacteria; o__Pseudomonadales; f__Pseudomonadaceae; g__Pseudomonas |
| f07f577cee394aac234643beb51f3a28 | d__Bacteria; p__Proteobacteria; c__Alphaproteobacteria |
| 884647b522ef48acb57a64e71987a20f | d__Bacteria; p__Proteobacteria; c__Gammaproteobacteria; o__Pseudomonadales; f__Moraxellaceae; g__Enhydrobacter |
| 2ffdcd7a901d95a5cdfdfa7046f75335 | d__Bacteria; p__Proteobacteria; c__Gammaproteobacteria; o__Pseudomonadales; f__Moraxellaceae; g__Acinetobacter |
| deeb7c33ae40604e7d218dfd1211aa86 | d__Bacteria; p__Proteobacteria; c__Gammaproteobacteria; o__Enterobacteriales; f__Enterobacteriaceae; g__Rosenbergiella |
| 553b2dd4e3e99efaff8c925c7f14bc71 | d__Bacteria; p__Proteobacteria; c__Gammaproteobacteria; o__Enterobacteriales; f__Enterobacteriaceae; g__Raoultella |
| 2b3e0949f0335231276d82089e09bab1 | d__Bacteria; p__Actinobacteria; c__Actinobacteria; o__Micrococcales; f__Micrococcaceae; g__Glutamicibacter |
| 47a96ecd06c9b0e338552dfde789e941 | d__Bacteria; p__Proteobacteria; c__Gammaproteobacteria; o__Alteromonadales; f__Pseudoalteromonadaceae; g__Pseudoalteromonas |
| 3661a97dd3f6ebf126d3a0b7de849870 | d__Bacteria; p__Proteobacteria; c__Gammaproteobacteria; o__Vibrionales; f__Vibrionaceae; g__Photobacterium |
| 22effb138a706f3e274eccb0e9f59a71 | d__Bacteria; p__Actinobacteria; c__Actinobacteria; o__Micrococcales; f__Microbacteriaceae; g__Microbacterium |
| 5eba0797a369a0c0ed73d9d76d1ea755 | d__Bacteria; p__Proteobacteria; c__Gammaproteobacteria; o__Pseudomonadales; f__Moraxellaceae; g__Acinetobacter; s__uncultured bacterium |
| 0f57c64b10138a27b134f20580d3b716 | d__Bacteria; p__Actinobacteria; c__Actinobacteria; o__Propionibacteriales; f__Propionibacteriaceae; g__Propionibacterium; s__uncultured bacterium |
| f4943dd6810c8392435d9549175f6642 | d__Bacteria; p__Proteobacteria; c__Gammaproteobacteria; o__Vibrionales; f__Vibrionaceae; g__Vibrio; s__Vibrio porteresiae |
| 1e3bbf037ca5d81285cde44f11b127e4 | d__Bacteria; p__Firmicutes; c__Bacilli; o__Lactobacillales; f__Enterococcaceae; g__Enterococcus |
| bfa470764a664b737c23f71775073f8e | d__Bacteria; p__Proteobacteria; c__Alphaproteobacteria; o__Caulobacterales; f__Caulobacteraceae; g__Brevundimonas; s__uncultured bacterium |
| 9e030e43277a50c91feb13e2782fd10b | d__Bacteria; p__Actinobacteria; c__Actinobacteria; o__Micrococcales; f__Microbacteriaceae; g__Microbacterium |
| db66b4cdbeec2bf13c09c4dd4320d155 | d__Bacteria; p__Proteobacteria; c__Gammaproteobacteria; o__Enterobacteriales; f__Enterobacteriaceae; g__Serratia |
| 79071165a9c61c2a6408392b138dd31c | d__Bacteria; p__Firmicutes; c__Bacilli; o__Lactobacillales; f__Leuconostocaceae; g__Leuconostoc; s__Leuconostoc lactis |
| 6d5576f8714268e8bf9e6c16cb45a4a6 | d__Bacteria; p__Proteobacteria; c__Alphaproteobacteria; o__Rhizobiales; f__Rhizobiaceae; g__Rhizobium |
| 5a19be2cad8fad86243222aac032a8dd | d__Bacteria; p__Proteobacteria; c__Gammaproteobacteria; o__Pseudomonadales; f__Moraxellaceae; g__Acinetobacter |
| a2361801ec118da583bd9e01a64da23c | d__Bacteria; p__Proteobacteria; c__Gammaproteobacteria; o__Xanthomonadales; f__Xanthomonadaceae; g__Stenotrophomonas; s__Stenotrophomonas maltophilia |
| bb04b40a9034fc48c881843663fb665c | d__Bacteria; p__Firmicutes; c__Bacilli; o__Lactobacillales; f__Enterococcaceae; g__Enterococcus |
| 68b38de6a6e9efb10dba9d2f747bb4e8 | d__Bacteria; p__Bacteroidetes; c__Flavobacteriia; o__Flavobacteriales; f__Flavobacteriaceae; g__Zunongwangia |
| ce6e618d63cf538bef3e90e7e77941ed | d__Bacteria; p__Proteobacteria; c__Gammaproteobacteria; o__Enterobacteriales; f__Enterobacteriaceae; g__Raoultella |
| d3b6285396892be4048451fc684513d7 | d__Bacteria; p__Proteobacteria; c__Gammaproteobacteria; o__Pseudomonadales; f__Moraxellaceae; g__Acinetobacter; s__uncultured bacterium |
| b75472a118da184abc9004e4b142f7ad | d__Bacteria; p__Proteobacteria; c__Betaproteobacteria; o__Burkholderiales; f__Comamonadaceae; g__Comamonas; s__uncultured bacterium |
| 1c3657ff1187155b6795207920189a93 | d__Bacteria; p__Proteobacteria; c__Alphaproteobacteria; o__Sphingomonadales; f__Sphingomonadaceae; g__Sphingomonas |
| 121d3c13c307a09c50a3864a603a1e98 | d__Bacteria; p__Proteobacteria; c__Alphaproteobacteria; o__Rhizobiales; f__Rhizobiaceae; g__Rhizobium; s__uncultured bacterium |
| 7fae4aca68664aea24076e63ccbf409d | d__Bacteria; p__Firmicutes; c__Bacilli; o__Bacillales; f__Staphylococcaceae; g__Staphylococcus; s__uncultured bacterium |
| 91b486f1d8cb88890f2031a04de220ba | d__Bacteria; p__Proteobacteria; c__Alphaproteobacteria; o__Sphingomonadales; f__Sphingomonadaceae; g__Sphingomonas; s__uncultured bacterium |
| 66dc937044b0f8a5e6b0fad8a670f015 | d__Bacteria; p__Proteobacteria; c__Alphaproteobacteria; o__Rhizobiales; f__Rhizobiaceae; g__Rhizobium; s__Agrobacterium sp. H13-3 |
| 8b3cabc78adcf47638ec3a9b20678524 | d__Bacteria; p__Proteobacteria; c__Alphaproteobacteria; o__Rhizobiales; f__Methylobacteriaceae; g__Methylobacterium |
| 8bb4cbd0487cca7f680fd3c58b9b51a0 | d__Bacteria; p__Proteobacteria; c__Gammaproteobacteria; o__Xanthomonadales; f__Xanthomonadaceae; g__Xanthomonas; s__Xanthomonas axonopodis pv. punicae |
| 3ec10b5fc0d6a8d9245f251d8d55ea6c | d__Bacteria; p__Proteobacteria; c__Alphaproteobacteria; o__Rhizobiales; f__Rhizobiaceae; g__Rhizobium; s__Rhizobium radiobacter |
| 8fdf5a16043b4d7f5cd9b57da1d0718e | d__Bacteria; p__Bacteroidetes; c__Flavobacteriia; o__Flavobacteriales; f__Flavobacteriaceae; g__Leeuwenhoekiella |
| 871a7ef39c763dafb86cc95f494e5b4a | d__Bacteria; p__Bacteroidetes; c__Sphingobacteriia; o__Sphingobacteriales; f__Sphingobacteriaceae; g__Sphingobacterium |
| 1c92ddcc1c56895b304e76e1d9b4f9e6 | d__Bacteria; p__Firmicutes; c__Bacilli; o__Bacillales; f__Bacillaceae; g__Bacillus; s__Bacillus marisflavi |
| af466a42bc9f60c62dc0ed482d13eda7 | d__Bacteria; p__Proteobacteria; c__Gammaproteobacteria; o__Pseudomonadales; f__Moraxellaceae; g__Acinetobacter; s__uncultured bacterium |
| 7b5670219bc20a63ce97769ad8e86415 | d__Bacteria; p__Proteobacteria; c__Deltaproteobacteria; o__Myxococcales; f__Archangiaceae; g__Melittangium; s__Melittangium lichenicola |
| 207807a8539405f72f4f001521f6b896 | d__Bacteria; p__Proteobacteria; c__Gammaproteobacteria; o__Xanthomonadales; f__Xanthomonadaceae; g__Stenotrophomonas; s__Stenotrophomonas maltophilia |
| 4cf3653bb5b8e60db99ba675f9916dcc | d__Bacteria; p__Proteobacteria; c__Alphaproteobacteria; o__Rhizobiales; f__Methylobacteriaceae; g__Methylobacterium |
| 1242585f484eea26e872720abee32add | d__Bacteria; p__Proteobacteria; c__Gammaproteobacteria; o__Pseudomonadales; f__Pseudomonadaceae; g__Pseudomonas |
| 56ddf8932c928dfde5925aeb37db17a6 | d__Bacteria; p__Proteobacteria; c__Gammaproteobacteria; o__Alteromonadales; f__Alteromonadaceae; g__Alteromonas |
| 06c2cbddbe83f9d502dcf04b52501ee3 | d__Bacteria; p__Proteobacteria; c__Alphaproteobacteria; o__Sphingomonadales; f__Sphingomonadaceae; g__Sphingomonas; s__Sphingomonas endophytica |
| 8af0ce98f7aa673d9dfee7db6fefe83c | d__Bacteria; p__Proteobacteria; c__Gammaproteobacteria; o__Alteromonadales; f__Pseudoalteromonadaceae; g__Pseudoalteromonas; s__uncultured organism |
| 14357451bf60c7fcf679835bedfc32df | d__Bacteria; p__Proteobacteria; c__Gammaproteobacteria; o__Enterobacteriales; f__Enterobacteriaceae; g__Kosakonia |
| 5c53f7f93c22e9b73b70c76325c952d5 | d__Bacteria; p__Proteobacteria; c__Betaproteobacteria; o__Burkholderiales; f__Comamonadaceae; g__Comamonas; s__uncultured bacterium |
| 4ee7dc733ffcbd75e57d2121e6ffb3e7 | d__Bacteria; p__Proteobacteria; c__Gammaproteobacteria; o__Oceanospirillales; f__Oceanospirillaceae; g__Marinomonas |
| 4885bac3b86798e20b0960ca3f814a1d | d__Bacteria; p__Proteobacteria; c__Alphaproteobacteria |
| 6ab0b012877b7a3281e5b1ab1ed5bbc0 | d__Bacteria; p__Proteobacteria; c__Alphaproteobacteria; o__Sphingomonadales; f__Erythrobacteraceae; g__Erythrobacter |
| c28760ba4867f86440fe63ad774e0ca2 | d__Bacteria; p__Proteobacteria; c__Gammaproteobacteria; o__Oceanospirillales; f__Halomonadaceae; g__Salinicola; s__Salinicola halophilus |
| c4b6fc67c64dc2c3cb71da69dffbfaed | d__Bacteria; p__Actinobacteria; c__Actinobacteria; o__Micrococcales; f__Microbacteriaceae; g__Microbacterium |
| 1fe31185541e5bdad0d706be5b0c0929 | d__Bacteria; p__Bacteroidetes; c__Sphingobacteriia; o__Sphingobacteriales; f__Sphingobacteriaceae; g__Sphingobacterium; s__uncultured bacterium |
| b40ce7716d3a7cfd52331a0bd91394e1 | d__Bacteria; p__Actinobacteria; c__Actinobacteria; o__Micrococcales; f__Microbacteriaceae; g__Frigoribacterium; s__Frigoribacterium faeni |
| 040b82acf3d92c04907113ffcde9ecd9 | d__Bacteria; p__Proteobacteria; c__Gammaproteobacteria; o__Xanthomonadales; f__Xanthomonadaceae; g__Stenotrophomonas; s__uncultured Stenotrophomonas sp. |
| 1fe7e15b2e6f2b666a1546dd188732c3 | d__Bacteria; p__Proteobacteria; c__Alphaproteobacteria; o__Rhizobiales; f__Methylobacteriaceae; g__Methylobacterium; s__unidentified |
| fcdcfbc16f6eea1a0095c7357ea25584 | d__Bacteria; p__Proteobacteria; c__Alphaproteobacteria; o__Rhodobacterales; f__Rhodobacteraceae; g__Paracoccus |
| ac8b1655a0208583e567c51fd869bccc | d__Bacteria; p__Actinobacteria; c__Actinobacteria; o__Kineosporiales; f__Kineosporiaceae; g__Kineococcus |
| de14fdede0c25e7d0d7109aeca84d142 | d__Bacteria; p__Firmicutes; c__Bacilli; o__Bacillales; f__Family XII; g__Exiguobacterium |
| d0ba7e85d18a194ee876e08276986ecb | d__Bacteria; p__Actinobacteria; c__Actinobacteria; o__Micrococcales; f__Micrococcaceae; g__Micrococcus; s__Micrococcus luteus |
| 867163d09df1451ccdbc8aa5c6c411bf | d__Bacteria; p__Proteobacteria; c__Betaproteobacteria; o__Burkholderiales; f__Oxalobacteraceae; g__Massilia; s__uncultured bacterium |
| f415dd788d49fe04f5a42234aa8ee030 | d__Bacteria; p__Proteobacteria; c__Gammaproteobacteria; o__Vibrionales; f__Vibrionaceae; g__Vibrio |
| 0e00482c5271cfc2a7ce61bbafd3ed7e | d__Bacteria; p__Firmicutes; c__Bacilli; o__Lactobacillales; f__Aerococcaceae; g__Aerococcus |
| bc33e41b15be0ade6536916518fdeed5 | d__Bacteria; p__Proteobacteria; c__Alphaproteobacteria; o__Rhizobiales; f__Methylobacteriaceae; g__Methylobacterium |
| 81e038399926975d82c6101c2149274f | d__Bacteria; p__Firmicutes; c__Bacilli; o__Bacillales; f__Planococcaceae; g__Chryseomicrobium |
| 116efd2a89b2517cbb004e4840ab9a73 | d__Bacteria; p__Proteobacteria; c__Gammaproteobacteria; o__Xanthomonadales; f__Xanthomonadaceae; g__Stenotrophomonas; s__Stenotrophomonas chelatiphaga |
| ab540ebbaaa090e405d781906522bcc3 | d__Bacteria; p__Proteobacteria; c__Gammaproteobacteria; o__Alteromonadales; f__Pseudoalteromonadaceae; g__Pseudoalteromonas |
| f9542c46c7c8fdf9d9380c970398077f | d__Bacteria; p__Actinobacteria; c__Actinobacteria; o__Streptomycetales; f__Streptomycetaceae; g__Streptomyces |
| aa9f349463608a100e38c6eef9cc2b56 | d__Bacteria; p__Bacteroidetes; c__Flavobacteriia; o__Flavobacteriales; f__Flavobacteriaceae; g__Chryseobacterium |
| f3ec233fa7802f99ab567bd4fd87a28d | d__Bacteria; p__Proteobacteria; c__Alphaproteobacteria; o__Rhodospirillales; f__Rhodospirillaceae; g__Thalassospira; s__uncultured bacterium |
| 2d8cf5e3969daf3fc0f363e2d4e8f45a | d__Bacteria; p__Proteobacteria; c__Alphaproteobacteria; o__Sphingomonadales; f__Sphingomonadaceae; g__Sphingomonas |
| e4e1a2fc2ce3a2b81973c6ff1aad68f5 | d__Bacteria; p__Proteobacteria; c__Gammaproteobacteria; o__Pseudomonadales; f__Moraxellaceae; g__Acinetobacter; s__uncultured bacterium |
| 448f0b81f535182f77a0ab2a848bc957 | d__Bacteria; p__Proteobacteria; c__Alphaproteobacteria; o__Sphingomonadales; f__Sphingomonadaceae; g__Sphingopyxis; s__uncultured bacterium |
| 23f5f98e86b3cc7c9feaabdbe633ee5a | d__Bacteria; p__Firmicutes; c__Bacilli; o__Lactobacillales; f__Carnobacteriaceae; g__Marinilactibacillus; s__Marinilactibacillus psychrotolerans |
| 72164ff1f9905f8958b6c980b933c6e3 | d__Bacteria; p__Actinobacteria; c__Actinobacteria; o__Kineosporiales; f__Kineosporiaceae; g__Kineococcus |
| f6c9da2005033041fed6d56aed0e76de | d__Bacteria; p__Firmicutes; c__Bacilli; o__Bacillales; f__Bacillaceae; g__Bacillus; s__Bacillus subtilis |
| d9f7ef48429f1ccd8b035256e0889962 | d__Bacteria; p__Proteobacteria; c__Betaproteobacteria; o__Burkholderiales; f__Oxalobacteraceae; g__Herbaspirillum |
| 4560b041293a827517366b14d8797eb0 | d__Bacteria; p__Proteobacteria; c__Alphaproteobacteria; o__Sphingomonadales; f__Sphingomonadaceae; g__Sphingomonas; s__uncultured bacterium |
| 4b37e3dada2080f38f1e5e3b2fe31518 | d__Bacteria; p__Bacteroidetes; c__Flavobacteriia; o__Flavobacteriales; f__Flavobacteriaceae; g__Zunongwangia |
| 34ba3cc4cb127b65f7f37bef9e1fb49a | d__Bacteria; p__Bacteroidetes; c__Cytophagia; o__Cytophagales; f__Cytophagaceae; g__Hymenobacter; s__uncultured bacterium |
| 48cac34707b6666b9e54d89a9fb4bd7a | d__Bacteria; p__Proteobacteria; c__Gammaproteobacteria; o__Enterobacteriales; f__Enterobacteriaceae; g__Pantoea; s__Erwinia gerundensis |
| 74c10d45d5942740afeaa71387e9b159 | d__Bacteria; p__Proteobacteria; c__Gammaproteobacteria; o__Pseudomonadales; f__Pseudomonadaceae; g__Pseudomonas |
| 59dd1ded2b6a755a197bf9e575efdc49 | d__Bacteria; p__Actinobacteria; c__Actinobacteria; o__Micrococcales; f__Microbacteriaceae; g__Microbacterium |
| e10c2d31b346cac79d9d9a080a745657 | d__Bacteria; p__Proteobacteria; c__Alphaproteobacteria; o__Sphingomonadales; f__Sphingomonadaceae; g__Sphingomonas; s__uncultured bacterium |
| 7c3995be840bd266dafad91b448ab77f | d__Bacteria; p__Proteobacteria; c__Betaproteobacteria; o__Burkholderiales; f__Alcaligenaceae; g__Verticia |
| 0f964eab321aaabee971bc0289e514e2 | d__Bacteria; p__Actinobacteria; c__Actinobacteria; o__Micrococcales; f__Dermabacteraceae; g__Brachybacterium; s__Brachybacterium paraconglomeratum |
| 3556a347df5c22405b22532b4fd48028 | d__Bacteria; p__Bacteroidetes; c__Sphingobacteriia; o__Sphingobacteriales; f__Sphingobacteriaceae; g__Sphingobacterium |
| a630e8e3f6413ddb1a1365ec4f025821 | d__Bacteria; p__Proteobacteria; c__Betaproteobacteria; o__Burkholderiales; f__Alcaligenaceae; g__Achromobacter; s__Achromobacter xylosoxidans subsp. xylosoxidans |
| 9753de391c7ebf7f8e158ab4d0c78f78 | d__Bacteria; p__Proteobacteria; c__Alphaproteobacteria; o__Rhizobiales; f__Phyllobacteriaceae; g__Mesorhizobium |
| ff287a60abdedb122aed38aa05b5eba1 | d__Bacteria; p__Firmicutes; c__Bacilli; o__Bacillales; f__Planococcaceae; g__Lysinibacillus; s__Lysinibacillus sphaericus |
| 994da3beeed80cf5b095cefa3557b510 | d__Bacteria; p__Proteobacteria; c__Betaproteobacteria; o__Burkholderiales; f__Oxalobacteraceae; g__Massilia; s__uncultured bacterium |
| 14242397d25e0097ef28c23220376d52 | d__Bacteria; p__Proteobacteria; c__Gammaproteobacteria; o__Oceanospirillales; f__Halomonadaceae; g__Kushneria; s__Kushneria marisflavi |
| 1d8ebdaf2df5c44589561cb3b3d955c2 | d__Bacteria; p__Proteobacteria; c__Gammaproteobacteria; o__Thiotrichales; f__Piscirickettsiaceae; g__Methylophaga |
| c6f9e208e030fe187acc07af5791240c | d__Bacteria; p__Bacteroidetes; c__Sphingobacteriia; o__Sphingobacteriales; f__Sphingobacteriaceae; g__Sphingobacterium |
| 6c57632ba4aa0e63debaee63a92dd77c | d__Bacteria; p__Proteobacteria; c__Alphaproteobacteria; o__Sphingomonadales; f__Sphingomonadaceae; g__Sphingomonas; s__uncultured bacterium |
| 620e684a7ced4fbf68e880419ea30368 | d__Bacteria; p__Proteobacteria; c__Alphaproteobacteria; o__Sphingomonadales; f__Sphingomonadaceae; g__Sphingomonas |
| a39497e9edf3957b78f3b3662795f46a | d__Bacteria; p__Proteobacteria; c__Alphaproteobacteria; o__Rhodobacterales; f__Rhodobacteraceae; g__Paracoccus; s__uncultured bacterium |
| 5f673b19de03a87e678f171306c3bf30 | d__Bacteria; p__Actinobacteria; c__Actinobacteria; o__Micrococcales; f__Microbacteriaceae; g__Microbacterium |
| fdadb69dc4714458be6553a4be000e1a | d__Bacteria; p__Proteobacteria; c__Alphaproteobacteria; o__Sphingomonadales; f__Sphingomonadaceae; g__Sphingomonas; s__uncultured bacterium |
| 5fd682b04f89f5353035905d560acac5 | d__Bacteria; p__Firmicutes; c__Bacilli; o__Bacillales; f__Paenibacillaceae; g__Paenibacillus |
| 34aeaa5e210648f0bb7ca9a30767b67d | d__Bacteria; p__Proteobacteria; c__Alphaproteobacteria; o__Sphingomonadales; f__Sphingomonadaceae; g__Sphingomonas |
| 790a18d4779cefa4c551e52788170c2a | d__Bacteria; p__Actinobacteria; c__Actinobacteria; o__Corynebacteriales; f__Corynebacteriaceae; g__Corynebacterium 1; s__uncultured bacterium |
| dff5c26583b64514eee4e1521c23d94b | d__Bacteria; p__Proteobacteria; c__Alphaproteobacteria; o__Sphingomonadales; f__Erythrobacteraceae; g__Erythrobacter |
| a92af87558d95fba117476b3e0a7bc74 | d__Bacteria; p__Firmicutes; c__Bacilli; o__Bacillales; f__Bacillaceae; g__Bacillus; s__Bacillus circulans |
| 04edf706b360989d01aeab8503e287ba | d__Bacteria; p__Actinobacteria; c__Actinobacteria; o__Micrococcales; f__Brevibacteriaceae; g__Brevibacterium; s__uncultured bacterium |
| 4d1df615257252ea553416ee84e23475 | d__Bacteria; p__Proteobacteria; c__Alphaproteobacteria; o__Sphingomonadales; f__Sphingomonadaceae; g__Sphingomonas |
| 27b13e54ff45711d6e266a27dec89838 | d__Bacteria; p__Proteobacteria; c__Betaproteobacteria; o__Burkholderiales; f__Comamonadaceae; g__Comamonas; s__uncultured bacterium |
| 9920cde5db98bfb795919ef7bf924952 | d__Bacteria; p__Proteobacteria; c__Gammaproteobacteria; o__Enterobacteriales; f__Enterobacteriaceae; g__Morganella; s__uncultured organism |
| a65176b9233e6e25cfb64b98b2979a4e | d__Bacteria; p__Firmicutes; c__Bacilli; o__Bacillales; f__Planococcaceae; g__Planococcus; s__Planococcus salinarum |
| ded24aec6076da41bd556353e91a7d64 | d__Bacteria; p__Proteobacteria; c__Alphaproteobacteria; o__Rhizobiales; f__Hyphomicrobiaceae; g__Devosia |
| e17b4eaf1dd7851933375c02bc8286f1 | d__Bacteria; p__Proteobacteria; c__Gammaproteobacteria; o__Enterobacteriales; f__Enterobacteriaceae; g__Enterobacter |
| eeb3f5b780f1e5d91ac2ee510759fcfa | d__Bacteria; p__Proteobacteria; c__Alphaproteobacteria; o__Rhizobiales; f__Rhizobiaceae; g__Rhizobium |
| 0cde8047a15522eb1405222e3971ecd8 | d__Bacteria; p__Proteobacteria; c__Gammaproteobacteria; o__Alteromonadales; f__Alteromonadaceae |
| e44bf6f76e11f7b282fa94a37ff2d59e | d__Bacteria; p__Proteobacteria; c__Betaproteobacteria; o__Burkholderiales; f__Alcaligenaceae; g__Achromobacter |
| 2a1ac10a30c3d02c8a33129c5f60cf39 | d__Bacteria; p__Proteobacteria; c__Alphaproteobacteria; o__Rhizobiales; f__1174-901-12; g__norank; s__uncultured bacterium |
| ad21460a2a5523875fc1692eb0339583 | d__Bacteria; p__Bacteroidetes; c__Cytophagia; o__Cytophagales; f__Cytophagaceae; g__Hymenobacter |
| a539379793ba1b7acf12fa0a3a72072b | d__Bacteria; p__Proteobacteria; c__Alphaproteobacteria; o__Rhizobiales; f__Rhizobiaceae; g__Rhizobium |
| c7f1d1564b186d083078e599f88f2c45 | d__Bacteria; p__Actinobacteria; c__Actinobacteria; o__Micrococcales; f__Sanguibacteraceae; g__Sanguibacter |
| 13f79dd16979e0834357dd005d4c1909 | d__Bacteria; p__Proteobacteria |
| bbf3aff7b62d742a5bdde19f6d050564 | d__Bacteria; p__Proteobacteria; c__Alphaproteobacteria; o__Sphingomonadales; f__Sphingomonadaceae; g__Sphingomonas; s__uncultured bacterium |
| 73b56fcebbc826c19d790cd73e2f614a | d__Bacteria; p__Proteobacteria; c__Betaproteobacteria; o__Burkholderiales; f__Comamonadaceae; g__Pseudorhodoferax; s__uncultured bacterium |
| be80e04c642bc49246327eefdbc72e23 | d__Bacteria; p__Bacteroidetes; c__Flavobacteriia; o__Flavobacteriales; f__Flavobacteriaceae; g__Salegentibacter |
| 9264a7dfa910bb9e145e9eb899e7e29d | d__Bacteria; p__Actinobacteria; c__Actinobacteria; o__Micrococcales; f__Micrococcaceae; g__Kocuria; s__Kocuria rosea |
| 9279ef77bd5df269084f6717f7813c54 | d__Bacteria; p__Actinobacteria; c__Actinobacteria; o__Micrococcales; f__Microbacteriaceae; g__Amnibacterium |
| 17b92a0006bd006049bcaa6695cc729e | d__Bacteria; p__Proteobacteria; c__Gammaproteobacteria; o__Oceanospirillales; f__Oceanospirillaceae; g__Marinobacterium |
| 3fcc74e0656d92f92e5b95e2f2f4fc6f | d__Bacteria; p__Firmicutes; c__Bacilli; o__Bacillales; f__Paenibacillaceae; g__Paenibacillus |
| 70831532a36431b1b307f11bd258bf53 | d__Bacteria; p__Proteobacteria; c__Gammaproteobacteria; o__Pseudomonadales; f__Pseudomonadaceae; g__Pseudomonas; s__uncultured bacterium |
| 95b6b7dddac0da3ff68d370b13c8f444 | d__Bacteria; p__Proteobacteria; c__Alphaproteobacteria; o__Rhizobiales; f__Methylobacteriaceae; g__Methylobacterium |
| 0a6949db8277f486132435aa2e58b1c7 | d__Bacteria; p__Proteobacteria; c__Betaproteobacteria; o__Burkholderiales; f__Oxalobacteraceae; g__Massilia; s__uncultured bacterium |
| faa50b29243fb737c0adea1a1bcbff13 | d__Bacteria; p__Proteobacteria; c__Gammaproteobacteria; o__Pseudomonadales; f__Pseudomonadaceae; g__Pseudomonas; s__Pseudomonas putida |
| 482f6f74eaefd8b39d48af01f1eb32c5 | d__Bacteria; p__Proteobacteria; c__Betaproteobacteria; o__Burkholderiales; f__Comamonadaceae; g__Acidovorax |
| 6419e86c307f51382cf186635ba3bf83 | d__Bacteria; p__Bacteroidetes; c__Sphingobacteriia; o__Sphingobacteriales; f__Sphingobacteriaceae; g__Sphingobacterium; s__uncultured bacterium |
| 053d1a6124a9798528ae8117d6fd2ce8 | d__Bacteria; p__Proteobacteria; c__Gammaproteobacteria; o__Xanthomonadales; f__Xanthomonadaceae; g__Luteibacter; s__uncultured bacterium |
| f73476b9524c825b014a15139048d4fd | d__Bacteria; p__Proteobacteria; c__Alphaproteobacteria; o__Sphingomonadales; f__Sphingomonadaceae; g__Sphingomonas |
| 52966fb4ab138cfaa4689246f15147d6 | d__Bacteria; p__Proteobacteria; c__Alphaproteobacteria; o__Rhodobacterales; f__Rhodobacteraceae; g__Roseivivax; s__Roseivivax halodurans |
| 37020a198a70191d48a3b1a04a8a7ddd | d__Bacteria; p__Proteobacteria; c__Gammaproteobacteria; o__Pseudomonadales; f__Moraxellaceae; g__Acinetobacter; s__uncultured bacterium |
| 7547b027e618972aeb6875e80d3dd05f | d__Bacteria; p__Proteobacteria; c__Gammaproteobacteria; o__Oceanospirillales; f__Oceanospirillaceae; g__Marinomonas; s__Marinomonas aquimarina |
| 7a79c7a2194b6273b2e6322b1e5d1c46 | d__Bacteria; p__Proteobacteria; c__Alphaproteobacteria; o__Rhizobiales; f__Hyphomicrobiaceae; g__Devosia |
| 27791092ed70a89310e4175c4633324f | d__Bacteria; p__Proteobacteria; c__Alphaproteobacteria; o__Sphingomonadales; f__Sphingomonadaceae; g__Sphingomonas; s__Sphingomonas panni |
| d2bd103c44f8d2cd6d4da16aeafb4010 | d__Bacteria; p__Proteobacteria; c__Betaproteobacteria; o__Burkholderiales; f__Oxalobacteraceae; g__Massilia; s__uncultured bacterium |
| 2a5b2d59fe8a852862122a216811bba8 | d__Bacteria; p__Actinobacteria; c__Actinobacteria; o__Corynebacteriales; f__Dietziaceae; g__Dietzia |
| f3da7c012fc2d102c45490d6bc4c04dc | d__Bacteria; p__Proteobacteria; c__Gammaproteobacteria; o__Oceanospirillales; f__Oceanospirillaceae; g__Motiliproteus |
| 3e7899101d07b4fb98e50abd9658f298 | d__Bacteria; p__Firmicutes; c__Bacilli; o__Bacillales; f__Bacillaceae; g__Bacillus |
| 9bbdbd0e4980c892b3113bf4f3de2dbb | d__Bacteria; p__Proteobacteria; c__Alphaproteobacteria; o__Sphingomonadales; f__Erythrobacteraceae; g__Altererythrobacter; s__uncultured bacterium |
| 8c55249af870076a58dfd2014d936ce5 | d__Bacteria; p__Actinobacteria; c__Actinobacteria; o__Kineosporiales; f__Kineosporiaceae; g__Pseudokineococcus |
| 98ecb85de113436a7b090e7d4fd86a52 | d__Bacteria; p__Proteobacteria; c__Gammaproteobacteria; o__Xanthomonadales; f__Xanthomonadaceae; g__Stenotrophomonas; s__Stenotrophomonas rhizophila |
| acd40da7e7aca139f493477d3f65ccfb | d__Bacteria; p__Proteobacteria; c__Alphaproteobacteria; o__Rhodospirillales; f__Acetobacteraceae; g__Roseomonas |
| 9150ab27ce73417c7ec7b0ddec5e9e00 | d__Bacteria; p__Proteobacteria; c__Alphaproteobacteria; o__Caulobacterales; f__Caulobacteraceae; g__Brevundimonas; s__uncultured bacterium |
| d030182a766a1a738a3fa4bd0d2585ea | d__Bacteria; p__Proteobacteria; c__Alphaproteobacteria; o__Rhizobiales; f__Bradyrhizobiaceae; g__Bosea; s__uncultured organism |
| baa78a2f05376d180fa2d2509dd04d92 | d__Bacteria; p__Bacteroidetes; c__Flavobacteriia; o__Flavobacteriales; f__Flavobacteriaceae; g__Mesonia; s__Mesonia algae |
| f024f82067562c7681fa38132a9a6148 | d__Bacteria; p__Proteobacteria; c__Alphaproteobacteria; o__Sphingomonadales; f__Sphingomonadaceae; g__Sphingomonas; s__Sphingomonas dokdonensis |
| 97560cf0968bcbdcb4040571eb880a22 | d__Bacteria; p__Bacteroidetes; c__Cytophagia; o__Cytophagales; f__Flammeovirgaceae; g__Marivirga |
| 77edf0edb6530bbafeb21f0eadfe74a1 | d__Bacteria; p__Proteobacteria; c__Gammaproteobacteria; o__Pseudomonadales; f__Moraxellaceae; g__Psychrobacter |
| 500a1ea6beb15512b2164b9d0fd943a9 | d__Bacteria; p__Firmicutes; c__Bacilli; o__Bacillales; f__Staphylococcaceae; g__Macrococcus; s__uncultured bacterium |
| 48730efbc3b2a8d11a45db3849d0a8de | d__Bacteria; p__Proteobacteria; c__Alphaproteobacteria; o__Rhodobacterales; f__Rhodobacteraceae; g__Roseivivax |
| 4db220263bf2dff5bc18db8e9dbae4f2 | d__Bacteria; p__Proteobacteria; c__Alphaproteobacteria; o__Rhizobiales; f__Aurantimonadaceae; g__Aureimonas |
| 60012f2d933d339e5b51c3ad9b897c20 | d__Bacteria; p__Bacteroidetes; c__Flavobacteriia; o__Flavobacteriales; f__Flavobacteriaceae; g__Salegentibacter |
| 311c5d26ce8bfa27212f87ba4f19890b | d__Bacteria; p__Actinobacteria; c__Actinobacteria; o__Corynebacteriales; f__Corynebacteriaceae; g__Corynebacterium 1; s__uncultured bacterium |
| 0b9bf823140965340b6a3a669199cc6e | d__Bacteria; p__Bacteroidetes; c__Flavobacteriia; o__Flavobacteriales; f__Flavobacteriaceae; g__Neptunitalea |
| ecb3fab8b9a70930c8335de608c39ffc | d__Bacteria; p__Firmicutes; c__Clostridia; o__Clostridiales; f__Defluviitaleaceae; g__Defluviitaleaceae UCG-011; s__uncultured bacterium |
| 482370f47a665e78f0123d03753e75ec | d__Bacteria; p__Bacteroidetes; c__Flavobacteriia; o__Flavobacteriales; f__Flavobacteriaceae; g__Salegentibacter |
| 30da6112c5625739efd0ca0cd2ab853c | d__Bacteria; p__Proteobacteria; c__Gammaproteobacteria; o__Pseudomonadales; f__Pseudomonadaceae; g__Pseudomonas |
| a05a309c3968d5cd085cf1634974d341 | d__Bacteria; p__Actinobacteria; c__Actinobacteria; o__Micrococcales; f__Micrococcaceae; g__Kocuria; s__uncultured bacterium |
| 2e93d4f9e80fe1d8b6fd09b4aa0d5bd7 | d__Bacteria; p__Actinobacteria; c__Actinobacteria; o__Micrococcales; f__Microbacteriaceae; g__Plantibacter |
| 5ddb39e5755c56b038c40b12be9b7450 | d__Bacteria; p__Proteobacteria; c__Gammaproteobacteria; o__Enterobacteriales; f__Enterobacteriaceae; g__Pantoea |
| c3ed0b48fccf41993442c3280544ca48 | d__Bacteria; p__Proteobacteria; c__Gammaproteobacteria; o__Thiotrichales; f__Piscirickettsiaceae; g__Methylophaga; s__uncultured bacterium |
| 04fccdfd8c552bf604d8bae1492f6f65 | d__Bacteria; p__Proteobacteria; c__Betaproteobacteria; o__Burkholderiales; f__Comamonadaceae; g__Variovorax |
| 0260b087d043125f989b4da9d9f5306a | d__Bacteria; p__Proteobacteria; c__Gammaproteobacteria; o__Alteromonadales; f__Alteromonadaceae; g__Alteromonas |
| ebc3bada5e9f2bda4c532a9fd1520a92 | d__Bacteria; p__Proteobacteria; c__Alphaproteobacteria; o__Sphingomonadales; f__Sphingomonadaceae; g__Sphingomonas |
| 34af23d2c92baa6859b12577ae2b4f25 | d__Bacteria; p__Bacteroidetes; c__Flavobacteriia; o__Flavobacteriales; f__Flavobacteriaceae; g__Flavobacterium; s__Flavobacterium johnsoniae |
| 897d4de9017262c88a4edc6cb9be7b43 | d__Bacteria; p__Proteobacteria; c__Alphaproteobacteria; o__Rhizobiales; f__Hyphomicrobiaceae; g__Devosia; s__uncultured bacterium |
| bc7c8b97a68ac082cd4c35d35fc71c0c | d__Bacteria; p__Actinobacteria; c__Actinobacteria; o__Kineosporiales; f__Kineosporiaceae; g__Quadrisphaera; s__uncultured bacterium |
| ad62b703d5754e92e3d862113521c568 | d__Bacteria; p__Proteobacteria; c__Gammaproteobacteria; o__Cellvibrionales; f__Cellvibrionaceae; g__Gilvimarinus; s__uncultured bacterium |
| 5b1984a6c2eae064d601c8a36f5b5781 | d__Bacteria; p__Bacteroidetes; c__Flavobacteriia; o__Flavobacteriales; f__Flavobacteriaceae; g__Bizionia; s__uncultured bacterium |
| c642ac89f2a27df23dec41ab128e1683 | d__Bacteria; p__Proteobacteria; c__Alphaproteobacteria; o__Sphingomonadales; f__Sphingomonadaceae; g__Sphingomonas |
| 6fdbaf8fd5b4b9ae2bba8a8d1fa32435 | d__Bacteria; p__Proteobacteria; c__Gammaproteobacteria; o__Thiotrichales; f__Piscirickettsiaceae; g__Methylophaga |
| 219f20f05efad9cde413caafa5602721 | d__Bacteria; p__Actinobacteria; c__Actinobacteria; o__Micrococcales; f__Microbacteriaceae; g__Okibacterium; s__uncultured bacterium |
| 0a65fb16c6ec99827822551a981ae483 | d__Bacteria; p__Proteobacteria; c__Betaproteobacteria; o__Burkholderiales; f__Alcaligenaceae; g__Verticia |
| fb7019b56779cfa8f534089fa39b2a4e | d__Bacteria; p__Proteobacteria; c__Gammaproteobacteria; o__Oceanospirillales; f__Halomonadaceae; g__Halomonas |
| d86f882fe1f2cf96436c21ac628e60da | d__Bacteria; p__Actinobacteria; c__Actinobacteria; o__Kineosporiales; f__Kineosporiaceae; g__Pseudokineococcus; s__Pseudokineococcus marinus |
| 0746e6441cb7d0901f1243a74d234cf6 | d__Bacteria; p__Actinobacteria; c__Actinobacteria; o__Propionibacteriales; f__Nocardioidaceae; g__Nocardioides; s__uncultured bacterium |
| 104f53eff0c6ffa28b64466ae24d7a62 | d__Bacteria; p__Proteobacteria; c__Betaproteobacteria; o__Burkholderiales; f__Oxalobacteraceae; g__Massilia; s__uncultured bacterium |
| 9f132ba06ede1cbb28442855a81b89c5 | d__Bacteria; p__Proteobacteria; c__Betaproteobacteria; o__TRA3-20; f__norank; g__norank; s__uncultured bacterium |
| 86adf69fa516033d9b6995bb07dff125 | d__Bacteria; p__Proteobacteria; c__Betaproteobacteria; o__Burkholderiales; f__Comamonadaceae; g__Acidovorax; s__uncultured bacterium |
| bb09435895c39ec90991fa9403ae6f39 | d__Bacteria; p__Actinobacteria; c__Actinobacteria; o__Micrococcales; f__Promicromonosporaceae; g__Cellulosimicrobium |
| cbc474de437abc4f8bf6138227a72e53 | d__Bacteria; p__Proteobacteria; c__Gammaproteobacteria; o__Pseudomonadales; f__Moraxellaceae; g__Acinetobacter; s__uncultured bacterium |
| 453bfe17202cd23585a5e9501967fa0f | d__Bacteria; p__Bacteroidetes; c__Cytophagia; o__Cytophagales; f__Cytophagaceae; g__Hymenobacter; s__uncultured bacterium |
| 2303e24857c584e662c841ef9da16413 | d__Bacteria; p__Bacteroidetes; c__Flavobacteriia; o__Flavobacteriales; f__Flavobacteriaceae; g__Gramella |
| 2302b01f4a651dd3bb13feabf1171b9a | d__Bacteria; p__Bacteroidetes; c__Sphingobacteriia; o__Sphingobacteriales; f__Saprospiraceae; g__Lewinella |
| 8a00aa5eeda16bdfc90c55e3904a8a2e | d__Bacteria; p__Actinobacteria; c__Actinobacteria; o__Micrococcales; f__Microbacteriaceae; g__Leucobacter |
| e53971bfd2178fdc8aeb0797fbb3d143 | d__Bacteria; p__Firmicutes; c__Bacilli; o__Bacillales; f__Bacillaceae; g__Bacillus; s__Bacillus vietnamensis |
| 104063edf571e335254c6cde4d76130a | d__Bacteria; p__Actinobacteria; c__Actinobacteria; o__Corynebacteriales; f__Nocardiaceae; g__Rhodococcus |
| 6f2e4007ad4532dc3382f4f14c03aabb | d__Bacteria; p__Proteobacteria; c__Gammaproteobacteria; o__Cellvibrionales; f__Cellvibrionaceae; g__Marinimicrobium; s__Marinimicrobium agarilyticum DSM 16975 |
| a4824184ed580622afae1cf39ddc1f52 | d__Bacteria; p__Proteobacteria; c__Alphaproteobacteria; o__Rhizobiales; f__Bradyrhizobiaceae; g__Rhodopseudomonas; s__uncultured bacterium |
| 688e5c5b71f0a88457273bca23114e8f | d__Bacteria; p__Proteobacteria; c__Alphaproteobacteria; o__Sphingomonadales; f__Erythrobacteraceae; g__Erythrobacter |
| 19e25375358d29bdea6003ec468ededc | d__Bacteria; p__Proteobacteria; c__Alphaproteobacteria; o__Rhodobacterales; f__Rhodobacteraceae; g__norank |
| e3f273ed8d80a1d601564cca2132ea5e | d__Bacteria; p__Proteobacteria; c__Alphaproteobacteria; o__Rhodobacterales; f__Rhodobacteraceae; g__Palleronia |
| d28ca65130518639d99563d5fa191156 | d__Bacteria; p__Proteobacteria; c__Alphaproteobacteria; o__Sphingomonadales; f__Erythrobacteraceae; g__Croceicoccus |
| f0895fef17befed190910a6dcb862de3 | d__Bacteria; p__Bacteroidetes; c__Flavobacteriia; o__Flavobacteriales; f__Flavobacteriaceae; g__Chryseobacterium |
| 1f0ac9257e63a5c4d2e0ddd864bed249 | d__Bacteria; p__Proteobacteria; c__Gammaproteobacteria; o__Alteromonadales; f__Alteromonadaceae; g__Marinobacter; s__uncultured bacterium |
| 8f253af12493257ada41b9490360fa39 | d__Bacteria; p__Proteobacteria; c__Alphaproteobacteria; o__Sphingomonadales; f__Erythrobacteraceae; g__Altererythrobacter |
| 99871fbfe1710cd7464a9313a47e5b62 | d__Bacteria; p__Proteobacteria; c__Alphaproteobacteria; o__Rhizobiales; f__Rhizobiaceae; g__Rhizobium |
| 1aaad1ca2640bf5793c568edcb77e68b | d__Bacteria; p__Firmicutes; c__Bacilli; o__Bacillales; f__Listeriaceae; g__Brochothrix |
| 7713cbd56527e047d15abd5ccd80e9f2 | d__Bacteria; p__Proteobacteria; c__Gammaproteobacteria; o__Cellvibrionales; f__Cellvibrionaceae; g__Cellvibrio |
| cf622b40c5a2bf97691e671da312f8ff | d__Bacteria; p__Proteobacteria; c__Gammaproteobacteria; o__Oceanospirillales; f__Saccharospirillaceae; g__Saccharospirillum; s__uncultured bacterium |
| f049ef85485d87612156bb784b062b0d | d__Bacteria; p__Proteobacteria; c__Gammaproteobacteria; o__Oceanospirillales; f__Oceanospirillaceae; g__Marinomonas; s__uncultured bacterium |
| ee65444f41bcbaf1b9d6c296e4e98811 | d__Bacteria; p__Proteobacteria; c__Alphaproteobacteria; o__Rhizobiales; f__Rhizobiaceae; g__Rhizobium |
| 1be325971f5fc2ee619c8fab90ed9aae | d__Bacteria; p__Proteobacteria; c__Gammaproteobacteria; o__Oceanospirillales; f__Oceanospirillaceae; g__Nitrincola; s__uncultured bacterium |
| 1ad9493bdc98593338fd710c725abc24 | d__Bacteria; p__Bacteroidetes; c__Sphingobacteriia; o__Sphingobacteriales; f__Sphingobacteriaceae; g__Nubsella; s__uncultured bacterium |
| 392b8dcdb6a207e4a958f2b789206dc2 | d__Bacteria; p__Bacteroidetes; c__Flavobacteriia; o__Flavobacteriales; f__Flavobacteriaceae; g__Flavimarina; s__uncultured Cytophaga sp. |
| 23e565640eb9b005be4ca750eb91bc90 | d__Bacteria; p__Bacteroidetes; c__Flavobacteriia; o__Flavobacteriales; f__Cryomorphaceae; g__Brumimicrobium |
| 819147602f1646b04c88d91e3ebef8e4 | d__Bacteria; p__Bacteroidetes; c__Cytophagia; o__Cytophagales; f__Flammeovirgaceae; g__Tunicatimonas |
| 55d23996dca104adef6a522614d8a591 | d__Bacteria; p__Proteobacteria; c__Alphaproteobacteria; o__Rhizobiales; f__Rhizobiaceae; g__Rhizobium; s__uncultured bacterium |
| d038d2963462ed29d8cc0ba609dedcd0 | d__Bacteria; p__Bacteroidetes; c__Sphingobacteriia; o__Sphingobacteriales; f__Saprospiraceae; g__Lewinella |
| 132a3da64bc1568c8fd4648ff4741168 | d__Bacteria; p__Bacteroidetes; c__Flavobacteriia; o__Flavobacteriales; f__Flavobacteriaceae; g__Salinimicrobium |
| 0a7d5fdb20d2e81e8f43e0577d0697de | d__Bacteria; p__Firmicutes; c__Bacilli; o__Bacillales; f__Bacillaceae; g__Bacillus |
| 3b24abaf162d195c8bf2fc489138af0f | d__Bacteria; p__Actinobacteria; c__Actinobacteria; o__Corynebacteriales; f__Mycobacteriaceae; g__Mycobacterium |
| 2b3297c79f2e2019daee81f0f8ea8662 | d__Bacteria; p__Proteobacteria; c__Alphaproteobacteria; o__Sphingomonadales; f__Sphingomonadaceae; g__Sphingomonas; s__uncultured bacterium |
| a3abc87159a8c5721b00a6977f03d362 | d__Bacteria; p__Actinobacteria; c__Actinobacteria; o__Micrococcales; f__Microbacteriaceae; g__Microbacterium |
| 4226d2eae45c83ddb35df0eca7a94486 | d__Bacteria; p__Firmicutes; c__Bacilli; o__Bacillales; f__Planococcaceae; g__Jeotgalibacillus |
| 13df78ae6283c240a3b624b2d6bcc31b | d__Bacteria; p__Actinobacteria; c__Actinobacteria; o__Pseudonocardiales; f__Pseudonocardiaceae; g__Pseudonocardia |
| ab6bc2de8ec557d99a2ebeee692f1b22 | d__Bacteria; p__Proteobacteria; c__Gammaproteobacteria; o__Oceanospirillales; f__Oceanospirillaceae; g__Marinospirillum |
| c2abafc904df531f0bb2a8f1cda68290 | d__Bacteria; p__Proteobacteria; c__Gammaproteobacteria; o__Alteromonadales; f__Alteromonadaceae; g__Marinobacter |
| b07c6c1c8093cc9f9b070e3eeb92e205 | d__Bacteria; p__Actinobacteria; c__Actinobacteria; o__Micrococcales; f__Beutenbergiaceae; g__Salana |
| 305ddaf732f445d4417a44e0100b461f | d__Bacteria; p__Proteobacteria; c__Alphaproteobacteria; o__Rhizobiales; f__Aurantimonadaceae; g__Fulvimarina; s__Fulvimarina pelagi |
| 20185bd1cef0c203738b906fed0e120c | d__Bacteria; p__Cyanobacteria; c__Cyanobacteria; o__norank; f__norank; g__norank; s__uncultured bacterium |
| 0cc323f4618905eb387cd1074576e984 | d__Bacteria; p__Actinobacteria; c__Actinobacteria; o__Propionibacteriales; f__Nocardioidaceae; g__Nocardioides |
| 81b1536e7defdcd4af57a650791bff45 | d__Bacteria; p__Bacteroidetes; c__Sphingobacteriia; o__Sphingobacteriales; f__Sphingobacteriaceae; g__Pedobacter; s__uncultured bacterium |
| 9c9d7f84941fbaaa45473f85a4b4553b | d__Bacteria; p__Proteobacteria; c__Gammaproteobacteria; o__Xanthomonadales; f__Xanthomonadaceae; g__Luteibacter; s__uncultured bacterium |
| a034c13e89d7c9396d1a35cb3436e15a | d__Bacteria; p__Firmicutes; c__Bacilli; o__Lactobacillales; f__Leuconostocaceae; g__Weissella |
| 4442a9b56cd458c2d8c35fad5ad64e1c | d__Bacteria; p__Bacteroidetes; c__Sphingobacteriia; o__Sphingobacteriales; f__NS11-12 marine group; g__norank; s__uncultured bacterium |
| 204fa46bebe7f7bb68e2c3be2411881f | d__Bacteria; p__Bacteroidetes; c__Flavobacteriia; o__Flavobacteriales; f__Flavobacteriaceae; g__Leeuwenhoekiella |
| 3dde18eeaa2c146b6b3ba83afe2d38d2 | d__Bacteria; p__Actinobacteria; c__Acidimicrobiia; o__Acidimicrobiales; f__Acidimicrobiaceae; g__Ilumatobacter; s__uncultured bacterium |
| 9df9e2d8ea7cd00a90f49eb26e554aec | d__Bacteria; p__Proteobacteria; c__Alphaproteobacteria; o__Rhizobiales; f__Phyllobacteriaceae; g__Mesorhizobium; s__Mesorhizobium thiogangeticum |
| 5b2dcb6fb810f808c96efb2e4d77545a | d__Bacteria; p__Proteobacteria; c__Betaproteobacteria; o__Burkholderiales; f__Alcaligenaceae; g__Pigmentiphaga; s__uncultured bacterium |
| 8a77c5eb7101a980eeb924b72de7fe0e | d__Bacteria; p__Proteobacteria; c__Gammaproteobacteria; o__Oceanospirillales; f__Saccharospirillaceae; g__Saccharospirillum |
| c66fea4183a3851091d8eae7a8c63067 | d__Bacteria; p__Actinobacteria; c__Actinobacteria; o__Micrococcales; f__Promicromonosporaceae; g__Isoptericola |
| 74237a9a4ad2d505244c099c4060fe7a | d__Bacteria; p__Proteobacteria; c__Alphaproteobacteria; o__Rhizobiales; f__Hyphomicrobiaceae; g__Devosia; s__uncultured Devosia sp. |
| 8af66d2770f107d00558855b03fd18b3 | d__Bacteria; p__Proteobacteria; c__Betaproteobacteria; o__Burkholderiales; f__Burkholderiaceae; g__Burkholderia-Paraburkholderia; s__uncultured Burkholderia sp. |
| 3bbb9017bf41104d66cba71b934f2f30 | d__Bacteria; p__Bacteroidetes; c__Cytophagia; o__Cytophagales; f__Cytophagaceae; g__Hymenobacter; s__uncultured bacterium |
| 670e142367ecf0762a8b1d49f2718c56 | d__Bacteria; p__Bacteroidetes; c__Bacteroidia; o__Bacteroidales; f__Prolixibacteraceae; g__Sunxiuqinia; s__uncultured bacterium |
| ab480b30329e3587c61644e5e996b6dc | d__Bacteria; p__Proteobacteria; c__Alphaproteobacteria; o__Rhodospirillales; f__Acetobacteraceae; g__Belnapia |
| f9e82547c8091a7d6e2466737f96a87d | d__Bacteria; p__Proteobacteria; c__Alphaproteobacteria; o__Sphingomonadales; f__Erythrobacteraceae; g__Erythrobacter |
| 35057ed49046f9758d62e9087db59ef7 | d__Bacteria; p__Proteobacteria; c__Alphaproteobacteria; o__Sphingomonadales; f__Sphingomonadaceae; g__Novosphingobium; s__uncultured bacterium |
| 6191b33784d669b3b0631cdcad27dcd9 | d__Bacteria; p__Bacteroidetes; c__Cytophagia; o__Cytophagales; f__Flammeovirgaceae; g__Catalinimonas |
| 9aa12e95fdf9d36622fb259073fc4407 | d__Bacteria; p__Proteobacteria; c__Alphaproteobacteria; o__Rhodobacterales; f__Rhodobacteraceae; g__Loktanella |
| ee028d1bd99557a382a7577eb613c019 | d__Bacteria; p__Proteobacteria; c__Alphaproteobacteria; o__Sphingomonadales; f__Sphingomonadaceae; g__Sphingomonas |
| c300b02e21c53db7154853dc444e5503 | d__Bacteria; p__Bacteroidetes; c__Bacteroidetes Incertae Sedis; o__Order III; f__uncultured; g__norank |
| f451cc6f1d7f96b37f289238699cfb48 | d__Bacteria; p__Cyanobacteria; c__Cyanobacteria; o__uncultured; f__norank; g__norank; s__uncultured bacterium |
| cd317df2434e5e626b70322e935abb30 | d__Bacteria; p__Proteobacteria; c__Gammaproteobacteria; o__Alteromonadales; f__Idiomarinaceae; g__Idiomarina; s__uncultured bacterium |
| 856a18b5303ef51f257996100fcddfeb | d__Bacteria; p__Proteobacteria; c__Gammaproteobacteria; o__Oceanospirillales; f__Halomonadaceae; g__Halomonas |
| 7dc90ca0492202de0d4ee8c6cc1dbfa5 | d__Bacteria; p__Actinobacteria; c__Actinobacteria; o__Micrococcales; f__Demequinaceae; g__Demequina; s__Demequina aestuarii |
| 5cedf0289415f27dad7d261ec155aa38 | d__Bacteria; p__Proteobacteria; c__Gammaproteobacteria; o__Pseudomonadales; f__Pseudomonadaceae; g__Pseudomonas |
| 71303337ebe798e6fece860761b82564 | d__Bacteria; p__Proteobacteria; c__Alphaproteobacteria; o__Rhodospirillales; f__Rhodospirillaceae; g__uncultured |
| b0079dbab6b283f5f688302ab5764aeb | d__Bacteria; p__Bacteroidetes; c__Cytophagia; o__Cytophagales; f__Cyclobacteriaceae; g__Cyclobacterium |
| e93ff6ec76a524c5c26c9dcb8abf94b4 | d__Bacteria; p__Proteobacteria; c__Alphaproteobacteria; o__Rhodobacterales; f__Rhodobacteraceae; g__Paracoccus |
| 59d72daee40cd01c388cde8013cc5278 | d__Bacteria; p__Proteobacteria; c__Alphaproteobacteria; o__Sphingomonadales; f__Erythrobacteraceae; g__Altererythrobacter |
| 00dd7d86abde289b80e1f033c6181c49 | d__Bacteria; p__Actinobacteria; c__Coriobacteriia; o__Coriobacteriales; f__Coriobacteriaceae; g__Olsenella |
| 1050c5d436b0e4cc758761411118f323 | d__Bacteria; p__Bacteroidetes; c__Bacteroidia; o__Bacteroidia Incertae Sedis; f__Draconibacteriaceae; g__uncultured; s__uncultured bacterium |
| 0d755cfec04b481af42b493617ea9256 | d__Bacteria; p__Proteobacteria; c__Alphaproteobacteria; o__Rhodobacterales; f__Rhodobacteraceae; g__Sulfitobacter |
| db773d105e2816f3a360e7f96e8d9818 | d__Bacteria; p__Proteobacteria; c__Gammaproteobacteria; o__Alteromonadales; f__Alteromonadaceae; g__Catenovulum |
| 93b7f85660d6f7c17796db9ea314528d | d__Bacteria; p__Proteobacteria; c__Alphaproteobacteria; o__Rhodobacterales; f__Rhodobacteraceae; g__Pseudoroseicyclus |
| 3a650b94a116b18a63ca189944059c05 | d__Bacteria; p__Bacteroidetes; c__Bacteroidia; o__Bacteroidales; f__Bacteroidales S24-7 group; g__norank; s__uncultured bacterium |
| fabf1e19a1947f583ea319d87042400c | d__Bacteria; p__Proteobacteria; c__Gammaproteobacteria; o__Oceanospirillales; f__Alcanivoracaceae; g__Alcanivorax |
| 53340cbedcd5b76b63fd6649669edd90 | d__Bacteria; p__Actinobacteria; c__Actinobacteria; o__Kineosporiales; f__Kineosporiaceae; g__Quadrisphaera; s__uncultured bacterium |
| 18a184f72fbcc76bce72796fdc8aedb1 | d__Bacteria; p__Bacteroidetes; c__Sphingobacteriia; o__Sphingobacteriales; f__Saprospiraceae; g__Lewinella; s__Lewinella marina |
| eca005ca1823d3a7ed01fc8a5d1b2ca2 | d__Bacteria; p__Proteobacteria; c__Deltaproteobacteria; o__Myxococcales; f__Blfdi19; g__norank; s__uncultured bacterium |
| 3ce2827f086728ba6a876d74592f47c6 | Unclassified |

Table S3 The shared ASVs in the microbiome only between the herbivorous Milu (Père David's Deer (*Elaphurus davidianus*) and dietary plants in this study

| ASV ID | Taxonomy |
| --- | --- |
| 0728d88c9e11a3b2434be6089a8bc85c | d__Bacteria; p__Proteobacteria; c__Betaproteobacteria; o__Burkholderiales; f__Comamonadaceae |
| da146cfacc8a0cebe9df966ea638391d | d__Bacteria; p__Bacteroidetes; c__Flavobacteriia; o__Flavobacteriales; f__Flavobacteriaceae; g__Chryseobacterium; s__Chryseobacterium piscicola |
| 8e29415cec86ba74eef37b818a0e3ea6 | d__Bacteria; p__Proteobacteria; c__Betaproteobacteria; o__Burkholderiales; f__Comamonadaceae; g__Variovorax |
| 74d7e37ee00aa0a0cfa7aa18e0ec5262 | d__Bacteria; p__Actinobacteria; c__Actinobacteria; o__Streptomycetales; f__Streptomycetaceae; g__Streptomyces |
| b03122a05bc563dbfc4d438c7a3bd131 | d__Bacteria; p__Bacteroidetes; c__Sphingobacteriia; o__Sphingobacteriales; f__Sphingobacteriaceae; g__Pedobacter; s__uncultured bacterium |
| e170a59585b9fd093c641ee2289a972a | d__Bacteria; p__Bacteroidetes; c__Flavobacteriia; o__Flavobacteriales; f__Flavobacteriaceae; g__Flavobacterium |
| bd5cb4bb14acc410224ef45b839f58fe | d__Bacteria; p__Bacteroidetes; c__Flavobacteriia; o__Flavobacteriales; f__Flavobacteriaceae; g__Chryseobacterium |
| 07ec055636d96d1667136a8759154902 | d__Bacteria; p__Bacteroidetes; c__Cytophagia; o__Cytophagales; f__Cyclobacteriaceae; g__Algoriphagus; s__Algoriphagus locisalis |
| 5b6c290071726c7e1bae52c0238682d5 | d__Bacteria; p__Bacteroidetes; c__Flavobacteriia; o__Flavobacteriales; f__Flavobacteriaceae; g__Flavobacterium |
| 0ba5d52a2359006dbfd21a6303703c13 | d__Bacteria; p__Firmicutes; c__Clostridia; o__Clostridiales; f__Clostridiales vadinBB60 group; g__norank; s__uncultured bacterium |
| 50c364c2e51bb9f3755400893df349d7 | d__Bacteria; p__Bacteroidetes; c__Flavobacteriia; o__Flavobacteriales; f__Flavobacteriaceae; g__Flavobacterium; s__uncultured bacterium |
| 0ceb73f218698073312a6fd133b4ee6a | d__Bacteria; p__Proteobacteria; c__Gammaproteobacteria; o__Pseudomonadales; f__Pseudomonadaceae; g__Pseudomonas |
| 48fdc44f16baef2dc068c4df6958f467 | d__Bacteria; p__Bacteroidetes; c__Flavobacteriia; o__Flavobacteriales; f__Flavobacteriaceae; g__Flavobacterium |
| fbb3c427afaac731a991c9b931de8da2 | d__Bacteria; p__Proteobacteria; c__Betaproteobacteria; o__Burkholderiales; f__Alcaligenaceae; g__Verticia |
| db70e5eb457513f80954dc9a4098bb5b | d__Bacteria; p__Bacteroidetes; c__Flavobacteriia; o__Flavobacteriales; f__Flavobacteriaceae; g__Flavobacterium; s__uncultured bacterium |
| 30fe76a662647a306b06192038eaaf2c | d__Bacteria; p__Proteobacteria; c__Alphaproteobacteria; o__Rhodobacterales; f__Rhodobacteraceae; g__Albirhodobacter; s__uncultured bacterium |
| 14936c279af49fbe6af21879898fff16 | d__Bacteria; p__Bacteroidetes; c__Flavobacteriia; o__Flavobacteriales; f__Flavobacteriaceae; g__Chryseobacterium |
| d79604cd68dae2112da00afdfd56b56d | d__Bacteria; p__Proteobacteria; c__Betaproteobacteria; o__Burkholderiales; f__Oxalobacteraceae; g__Noviherbaspirillum; s__uncultured bacterium |
| 2e48b6dffbb1194cd877f80d68a22093 | d__Bacteria; p__Proteobacteria; c__Alphaproteobacteria; o__Rhizobiales; f__Aurantimonadaceae; g__Aureimonas |
| 559a842c817b6c95bf8a004e45cac4dd | d__Bacteria; p__Proteobacteria; c__Alphaproteobacteria; o__Rhodospirillales; f__Acetobacteraceae; g__Roseomonas |
| c4ac7dccf19d2c0d7c4a1c3b95bb3d41 | d__Bacteria; p__Bacteroidetes; c__Cytophagia; o__Cytophagales; f__Cytophagaceae; g__Dyadobacter; s__Dyadobacter koreensis |
| f04958dda9f195a7f9e621d5c131c31e | d__Bacteria; p__Bacteroidetes; c__Flavobacteriia; o__Flavobacteriales; f__Flavobacteriaceae; g__Flavobacterium; s__Flavobacterium flevense |
| f7ffd1f7597077e475ca9811146e7b2b | d__Bacteria; p__Bacteroidetes; c__Flavobacteriia; o__Flavobacteriales; f__Flavobacteriaceae; g__Flavobacterium; s__uncultured bacterium |


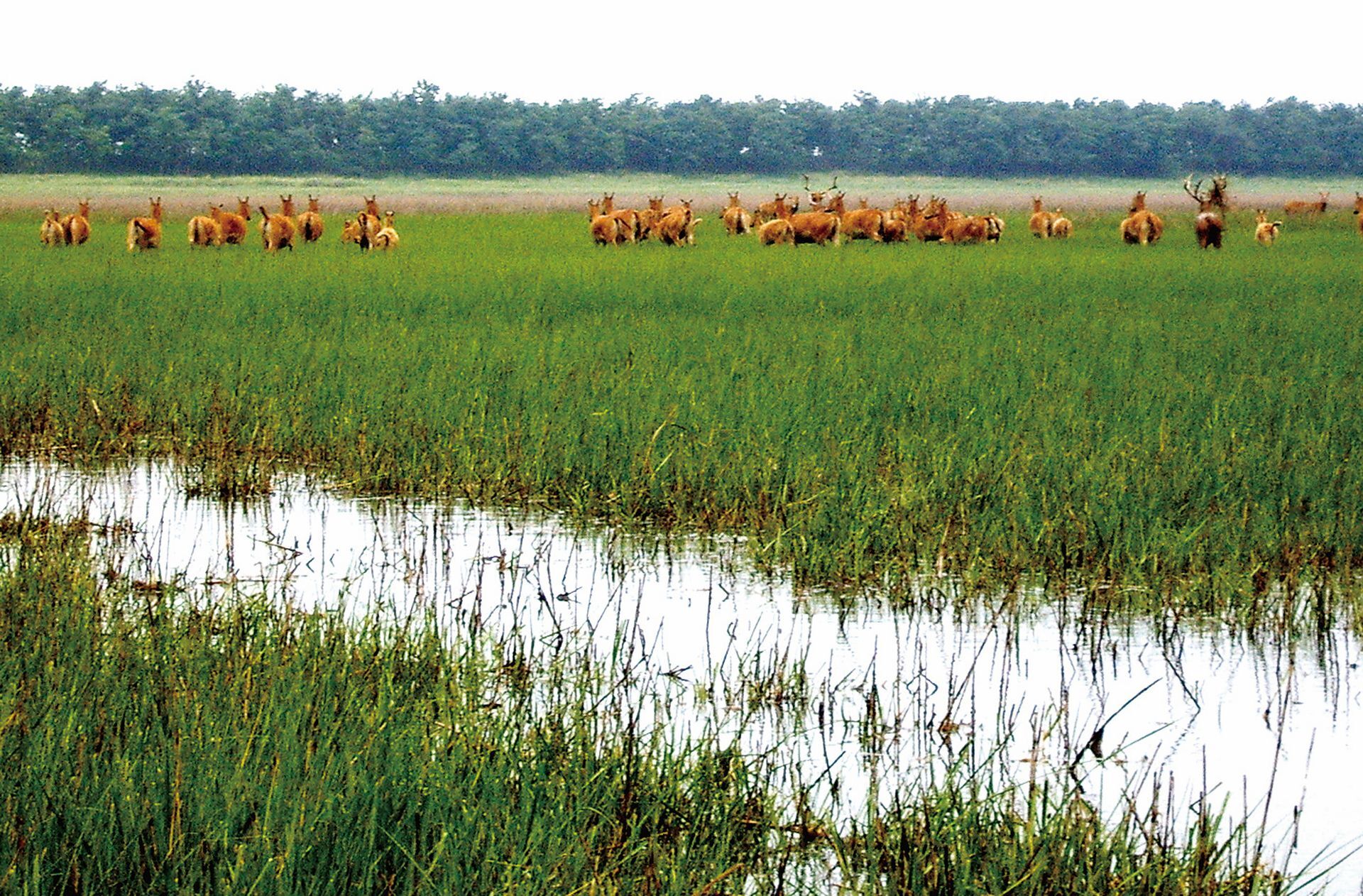


**Figure S1** The wild habitat for herbivorous insects (e.g., grasshoppers and locusts) and Milu (Père David’s deer *Elaphurus davidianus*) in Dafeng region. One of most dominant plants is *Spartina alterniflora*. The copyright belongs to Prof. Yuhua Ding.

**Figure S2** The shared ASVs among groups. Insects: leaf-eating grasshoppers and locusts in Dafeng region. Diet: dietary plants for the insects. Milu (Père David’s deer (*Elaphurus davidianus*)) in Dafeng region.

**Figure S3** Relative abundance of the ASVs shared only between the herbivorous insect (leaf-eating grasshoppers and locusts) gut microbiome and dietary plant symbiotic microbiome in Dafeng region
